# Nile Red Fluorescence: Where’s the Twist?

**DOI:** 10.1021/acs.jpcb.4c06048

**Published:** 2024-11-14

**Authors:** Camilla Gajo, Darya Shchepanovska, Jacob F. Jones, Gabriel Karras, Partha Malakar, Gregory M. Greetham, Olivia A. Hawkins, Caleb J. C. Jordan, Basile F. E. Curchod, Thomas A. A. Oliver

**Affiliations:** 1 School of Chemistry, Cantock’s Close, University of Bristol, Bristol BS8 1TS, U.K.; 2 Central Laser Facility, Science and Technology Facilities Council, Research Complex at Harwell, Rutherford Appleton Laboratory, Didcot, Oxfordshire OX11 0QX, U.K.

## Abstract

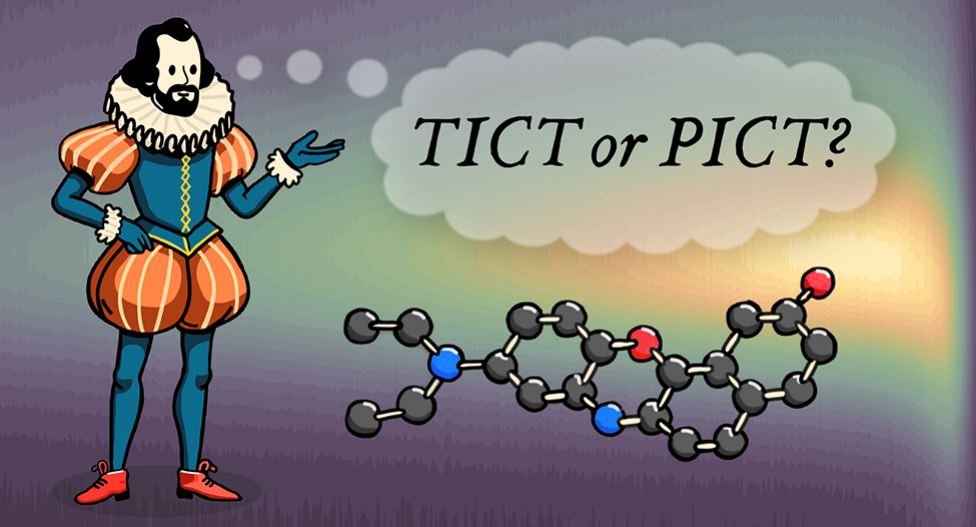

Nile Red is a fluorescent dye used extensively in bioimaging due to its strong solvatochromism. The photophysics underpinning Nile Red’s fluorescence has been disputed for decades, with some studies claiming that the dye fluoresces from two excited states and/or that the main emissive state is twisted and intramolecular charge-transfer (ICT) in character as opposed to planar ICT (PICT). To resolve these long-standing questions, a combined experimental and theoretical study was used to unravel the mechanism of Nile Red’s fluorescence. Time-resolved fluorescence measurements indicated that Nile Red emission occurs from a single excited state. Theoretical calculations revealed no evidence for a low-lying TICT state, with the S_1_ minimum corresponding to a PICT state. Ultrafast pump–probe spectroscopic data contained no signatures associated with an additional excited state involved in the fluorescence decay of Nile Red. Collectively, these data in polar and nonpolar solvents refute dual fluorescence in Nile Red and definitively demonstrate that emission occurs from a PICT state.

## 1 Introduction

Nile Red (9-(diethylamino)-5*H*-benzo[*a*]phenoxazin-5-one) is an important solvatochromic fluorescent dye that is widely used in bioimaging due to its remarkable environmental sensitivity. This renders the molecule an optimal candidate for reporting on the diverse environments encountered within complex biological systems, e.g., those that vary from highly polar to dense lipid conditions.^1−8^ Further, due to its biocompatibility, broad absorption spectrum, and appreciable fluorescence quantum yield, it is often used as a FRET marker to follow protein folding processes.^9−14^ Beyond biosensing, Nile Red has been used to detect microplastics in water^15,16^ and aid the detection of toxic gases.^17^ Despite its prevalent use in multiple fields, the photophysical origin of Nile Red’s fluorescence remains controversial. The debate primarily revolves around whether fluorescence occurs from a single excited state^18−20^ or two different electronic states (e.g., “dual fluorescence”)^21−23^ and the electronic character of the fluorescent state(s).

**Figure 1:**
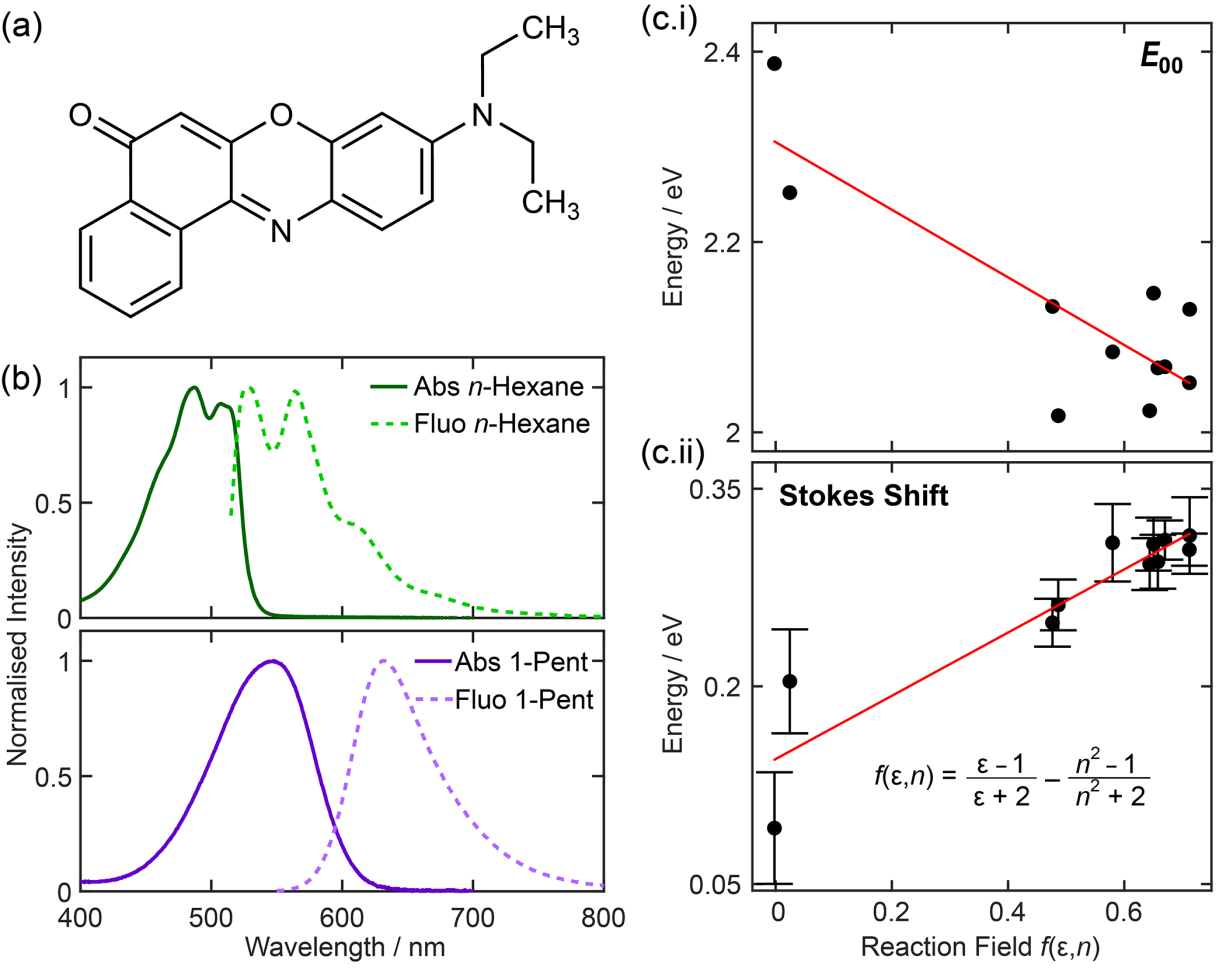
(a) Chemical structure of Nile Red. (b) Steady-state absorption and fluorescence spectra in solvents of different polarities: *n*-hexane and 1-pentanol. (c.i) *E*_00_ energy and (c.ii) Stokes shifts extracted from steady-state data as a function of reaction field, *f*(ε,*n*), for Nile Red in 12 different solvents. *E*_00_ was determined from the crossing point between the normalized absorption and fluorescence spectra to ensure consistency across all data sets.

The dye is composed of an extended π-conjugated system, with a terminal electron-donating diethylamino group; see the chemical structure shown in Figure 1a. Similarly to the excited-state dynamics of DMABN (4-(*N*,*N*-dimethylamino)benzonitrile),^24−26^ there is a long-standing debate on the role of the electron-donating amino group in the excited-state photophysics: Does twisting around the diethylamino moiety drive transfer between two different excited states of “locally excited” and charge-transfer character?^20−23,27−32^ Several theoretical and experimental studies have proposed that the molecule’s solvatochromism originates from a very strong excited-state dipole moment, which owes its magnitude to out-of-plane twisting of the terminal diethylamino group that enhances the charge-transfer between the diethylamino and carbonyl groups. Such excited states are frequently termed twisted intramolecular charge-transfer (TICT) states.^33^ Conversely, several time-dependent density functional theory (TDDFT) studies have shown that twisting around the electron-donating group is energetically unfavorable and advocate for a planar ICT (PICT) state.^18,20^ A subsequent theoretical study of Nile Red using multireference methods^23^ concluded that the excited-state minimum corresponded to a TICT state, and supported a dual fluorescence mechanism. Despite the numerous investigations to date, no consensus has emerged, and Nile Red is often cited as a TICT molecule in the wider literature.^32,34−37^

Using an array of experimental and theoretical techniques, many of which have not been previously applied to study the excited-state photophysics of Nile Red, we definitively show that the fluorescence of Nile Red originates from a single excited electronic state that has a significant intramolecular charge-transfer character. Our studies prove that the S_1_ fluorescent state is planar (e.g., PICT) and, counter to many prior studies,^22,23,28,29,31^ does not involve any twisting of the terminal diethylamino group. We show that the strong environmental sensitivity of the dye’s absorption and fluorescence maxima arises from the large permanent dipole moment associated with the ICT character of the S_1_ state and underpins the dye’s strong solvatochromism, a key property that makes Nile Red popular in bioimaging.^1−8^

## 2 Results and Discussion

The experimental and theoretical methods employed in this work and details of materials used are provided in the Supporting Information (SI). In nonpolar solvents, such as *n*-hexane (see Figure 1b), the fluorescence spectra of Nile Red have resolvable peaks that prior studies attributed to fluorescence from two different electronic states.^21,22^ However, as previously hypothesized,^18,20^ the peaks separated by ∼1280 cm^–1^ can alternatively be assigned to the vibronic structure that becomes increasingly inhomogeneously broadened in hydrogen-bonding solvents. Prior steady-state studies have established that the solvatochromism exhibited by Nile Red is strongly correlated with solvent polarity,^22,29,32,38^ as evident from the examples of steady-state absorption and fluorescence spectra shown in Figure 1b and in the SI (Figure S1). As the solvent polarity (parametrized by solvent dielectric and polarizability by a simple reaction field^39^) increases, the energy difference between the lowest excited (S_1_) and the ground (S_0_) electronic states of the molecule, *E*_00_, is reduced as apparent from the negative trend in Figure 1c.i. Further, increasing the solvent polarity also leads to a commensurate increase in the observed Stokes shift; see Figure 1c.ii. The polarity dependence of these two important parameters is consistent with an excited state that has a large permanent dipole moment.

Room temperature fluorescence excitation spectra in representative polar (ethanol) and nonpolar (*n*-hexane) solvents closely matched the respective absorption spectra (see Figure S2), indicating that only a single excited state contributes to the emission of Nile Red. Low-temperature (77 K) emission spectra in an ethanol glass (Figure S3) showed signatures of a vibrational structure, reminiscent of room temperature emission data collected in nonpolar solvents such as *n*-hexane (Figure S1) in contrast with the broad unresolved fluorescence spectrum recorded in room temperature ethanol solution. Prior temperature-dependent fluorescent studies of DMABN in ethanol and other polar solvents showed that the relative intensity of an emission band associated with the TICT state, only present in polar solvents, was strongly modulated with temperature.^40,41^ Our data do not exhibit the same phenomenon and are thus again entirely consistent with emission from a single electronic state.

**Figure 2:**
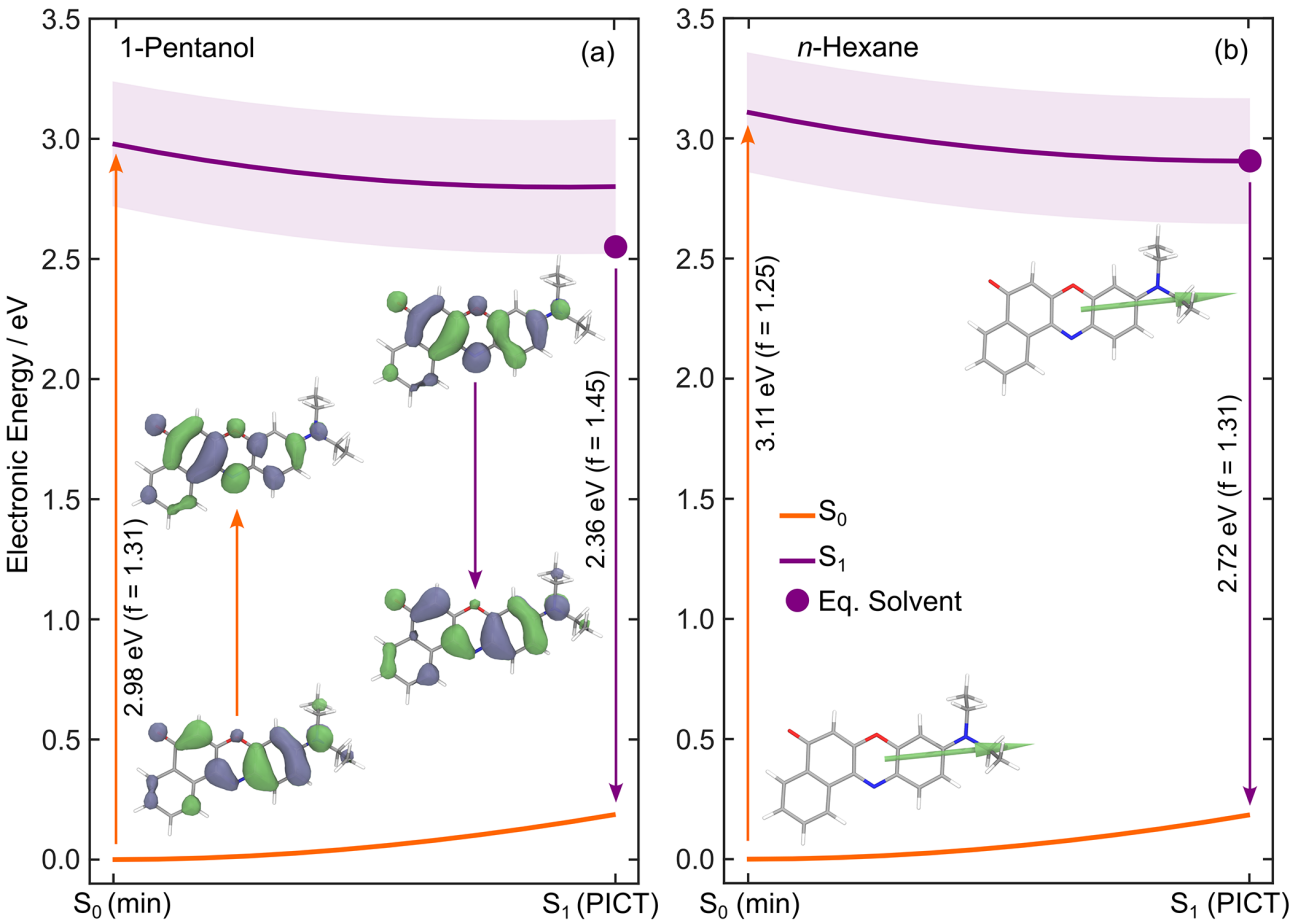
LIIC pathways connecting the ground-state optimized geometry, S_0_ (min), to the first excited optimized geometry, S_1_ (PICT), of Nile Red. Calculations were performed in an implicit solvent model for (a) 1-pentanol and (b) *n-*hexane. Insets in panel a depict the natural transition orbitals associated with absorption and fluorescence between the S_0_ and S_1_ states. In panel b, the transition dipole moments corresponding to the S_1_←S_0_ transition at S_0_ (min) and S_1_ (PICT) are overlaid on the respective minimum energy structures. The shaded purple area in both panels represents the magnitude of the S_1_ ← S_0_ oscillator strength along the LIIC pathway. The large purple dot in each panel corresponds to the solvent relaxed S_1_ minimum energy calculated using equilibrium IEFPCM. All calculations were performed with DFT/ωB97XD/def2-TZVPP and LR-TDDFT/TDA/ωB97XD/def2-TZVPP using a nonequilibrium IEFPCM solvent model.

Theory was used to explore the excited potential energy surfaces and the effect of twisting the terminal diethylamino group on excited-state potentials. *In primis*, minimum energy geometries were located on the ground (S_0_) and first excited (S_1_) electronic states using DFT (density functional theory) and LR-TDDFT/TDA (linear-response TDDFT within the Tamm–Dancoff approximation), with the ωB97XD functional, a def2-TZVPP basis set, and an implicit solvation model to mimic 1-pentanol and *n*-hexane solvents. Full details of the computational methods and benchmarking calculations are given in the SI. Interestingly, the S_1_ minimum energy geometry has an ICT character but does not exhibit any twist of the diethylamino group (see inset of Figure 2), echoing the conclusions of ref (18). We will henceforth describe the S_1_ state as a PICT state. The S_0_ and S_1_ minimum energy geometries are overall similar, with only minor variations in bond lengths of the molecular skeleton caused by the ^1^ππ* transition associated with the PICT state (see natural transition orbitals, NTOs, in the inset of Figure 2). Linear interpolation in internal coordinates (LIIC) pathways were constructed to connect these critical geometries and monitor the behavior of the potential energy surfaces along this path. As expected from the minor changes in molecular structure between the S_0_ and S_1_ optimized geometries (see Figure S6), the calculated LIIC pathways show a monotonic decrease of electronic energy in the first excited state from the Franck–Condon region to the S_1_ minimum, with no apparent barrier, in both *n*-hexane and 1-pentanol (see Figure 2). The electronic character of the S_1_ state strongly remains ICT along this relaxation pathway. This electronic character is defined by a shift of electron density from the diethylamino region of the molecule to the carbonyl group, as highlighted by the NTOs shown in Figure 2. The oscillator strength associated with the S_1_ ← S_0_ transition remains nearly constant along the LIIC (as depicted by the light purple shaded area along the S_1_ state potential curve in Figure 2). The calculations clearly indicate that the electronic character of the excited state from which excitation and emission occur should be the same, a view that is reinforced by the transition dipole moments (TDMs) shown in the inset of Figure 2b. This suggests that the initially prepared photoexcited electronic state is responsible for the vast majority of fluorescence in Nile Red, irrespective of the solvent environment.

This theoretical result was echoed in fluorescence anisotropy measurements in viscous solvent squalane. From these data, an initial rotational anisotropy value of +0.353 ± 0.002 was determined (see Figure S8), in agreement with prior reports.^42^ This value is close to the limiting case of +0.4 for parallel TDMs and supports the computational results, which return very close to parallel absorption and emission TDMs. Further, unlike DMABN,^43^ there was no wavelength dependence to the initial value of the rotational anisotropy for Nile Red, again pointing strongly to a single excited state being responsible for the molecule’s fluorescence.

Nile Red’s solvatochromism can be rationalized by the large ground- and S_1_ excited-state permanent dipole moments, which are calculated to be 9.4 and 11.1 D in *n*-hexane, and 11.8 and 15.1 D in 1-pentanol at the S_0_ and S_1_ optimized geometries, respectively, and in agreement with prior predictions.^44^ The stabilization of solvent equilibration at the S_1_ state minimum could also be estimated theoretically—see the difference between the LIIC energy at S_1_ (PICT) and the filled circle in Figure 2. As expected for a molecule with a large excited-state permanent dipole moment, the stabilization induced by solvent equilibration is far greater in polar solvents such as 1-pentanol than *n*-hexane. In the latter case, the estimated solvent relaxation is almost zero. These theoretical findings corroborate the experimentally determined Stokes shifts—see Figure 1c.

**Figure 3:**
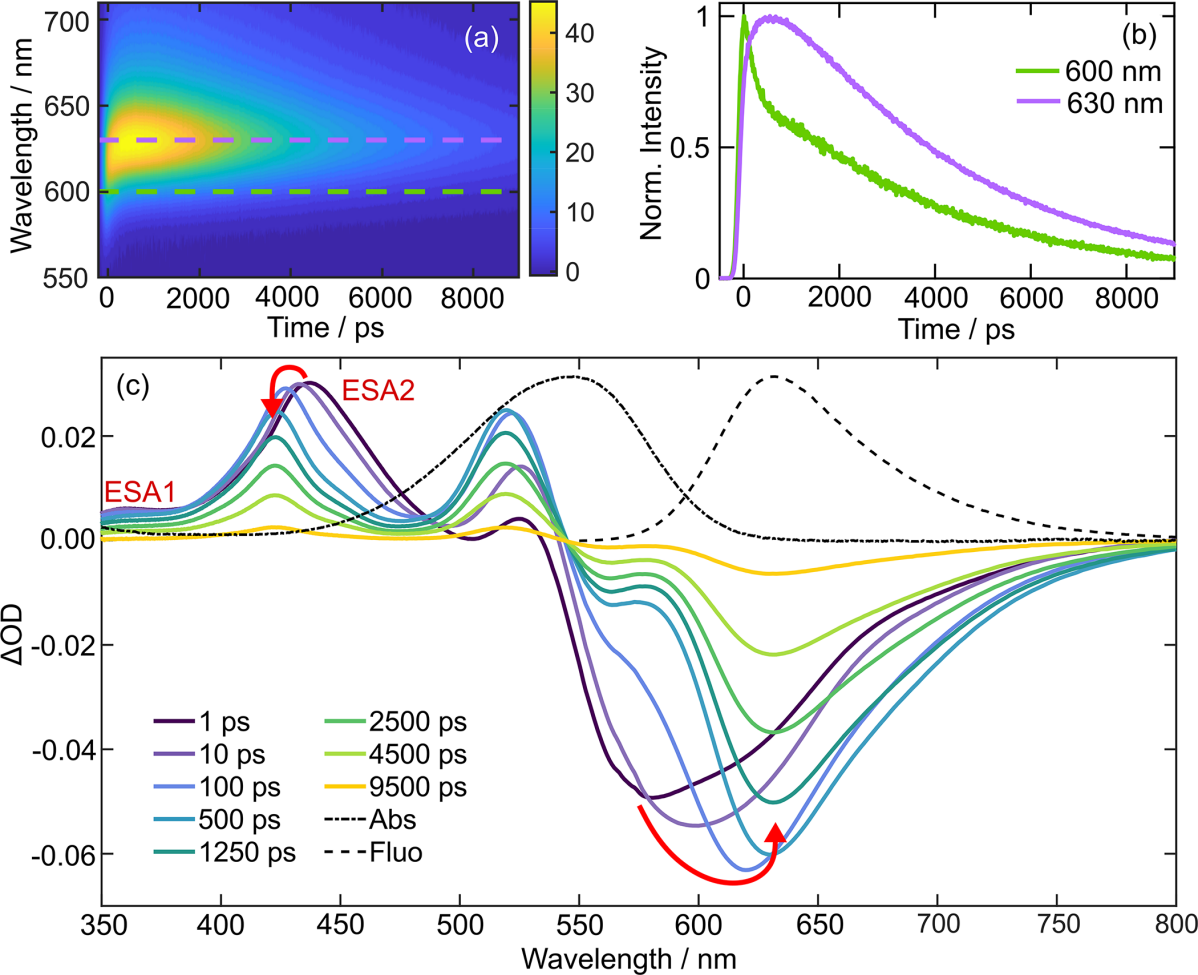
Time-resolved data of Nile Red in 1-pentanol following 550 nm photoexcitation. (a) WR-TCSPC false color contour map. (b) Fluorescence decays extracted from WR-TCSPC data at 600 and 630 nm. (c) Transient absorption data with overlaid red arrows highlighting the major spectral shifts.

Wavelength-resolved time-correlated single photon counting (WR-TCSPC) experiments were conducted to investigate whether the fluorescence lifetimes exhibited a wavelength dependence (see the SI for full experimental details).^45^Figure 3a shows WR-TCSPC data of Nile Red in 1-pentanol after 550 nm photoexcitation as a false-color contour plot. Within the first ∼750 ps, the fluorescence maximum red-shifts from ∼618 to 630 nm and subsequently decays with a 3.90 ± 0.18 ns time constant. Details of how the kinetics were extracted are given in the SI. The kinetics at 600 and 630 nm are shown in Figure 3b and exhibits different dynamics at ≤1.5 ns. While both fluorescence wavelengths share the same nanosecond relaxation, at wavelengths shorter than 615 nm, the kinetics tends toward a biexponential decay profile, whereas at longer wavelengths than 615 nm, a slow rise component is apparent followed by a monoexponential decay. The fluorescence lifetimes of Nile Red at *single* fluorescence wavelengths are reminiscent of those previously reported in similarly viscous propanol/glycerol mixtures;^21^ however, we have the full wavelength-resolved fluorescence dynamics and theoretical data to guide interpretation. Previously, the two different lifetimes were associated with fluorescence from two different electronic states.^21^ The fast decay component of the biexponential decay was assigned to fluorescence from a “locally excited” (LE) state and the nanosecond component evident across the whole wavelength range from the polarity sensitive ICT state. The slow rise component seen at longer wavelengths was attributed to a growth in the ICT population upon LE → ICT transfer. However, from the time evolution of the WR-TCSPC contour map presented in Figure 3b, it is apparent that the fluorescence maximum is instead red-shifting on a 250 ± 180 ps time scale, as expected for the dynamic Stokes shift in this solvent.^46^ Further, this interpretation is entirely consistent with our theoretical calculations that indicate that only one excited state is responsible for Nile Red’s fluorescence.

Similar signatures of a dynamic Stokes shift are apparent in WR-TCSPC measurements in other viscous hydrogen-bonding solvents such as benzyl alcohol and diethyl glycol (Figures S23 and S24). The red-shifting dynamics, however, was not observed for Nile Red in solutions of squalane, acetone, acetonitrile, dichloromethane (DCM), dimethyl sulfoxide (DMSO), ethanol, methanol, *n*-hexane, or toluene (see Figures S13 and S15–S22). The lack of apparent dynamic Stokes shift is reconciled by the weak polarity and/or low viscosity of the solvents: Excited-state solvation dynamics of photoexcited molecules in liquids is a multistep process,^47,48^ wherein, in nonviscous solvents (e.g., methanol, acetonitrile), the final step is significantly faster (e.g., 10s ps) than our TCSPC instrument response (∼180 ps). Further, the magnitude of solvent relaxation in nonpolar solvents (e.g., toluene, *n*-hexane, and squalane) is small as evident from the minor Stokes shift (see Figure 1) and the theoretically calculated solvent relaxation energy of Nile Red in *n*-hexane. We note that the apparent absence of a Stokes shift within these solvents is in accord with a prior TCSPC study of Nile Red in methanol.^49^

The photoexcited dynamics of Nile Red were explored using transient absorption (TA) and time-resolved infrared (TRIR) spectroscopy in a subset of the 12 solvents studied with WR-TCSPC to probe the sub-180 ps time dynamics and further investigate whether optically dark species, such as the TICT state, are formed but nonemissive.

Nile Red was photoexcited with 546 nm pump pulses, and the time-evolution probed at multiple pump–probe time delays, *t*, with a white light supercontinuum probe spanning 350–800 nm (see the SI for full experimental details). The TA spectra for Nile Red in 1-pentanol are displayed in Figure 3c for eight different pump–probe time delays. At UV/blue probe wavelengths, the TA spectra are dominated by two positive features corresponding to excited-state absorptions (ESAs) peaking at 380 and 437 nm, labeled ESA1 and ESA2, respectively. In the visible/near IR probe region, there is a broad negative feature between 530 and 800 nm assigned to stimulated emission (SE) from the S_1_ state, as it matches the steady-state fluorescence (dashed line) at *t* > 500 ps, i.e., once solvent reorganization is complete. The absence of a prominent ground-state bleach (GSB) feature in this region, expected based on the steady-state absorption spectrum (overlaid dot and dashed line), is curious, but we surmise that the GSB is dwarfed by a combination of overlapping ESA signals.

**Table 1:** Excited-State Solvation Time Constants for Nile Red Retrieved from Shifts in the Central Emission Frequency in WR-TCSPC and TA Data^a^

**solvent**	**WR-TCSPC/ps**	**TA (SE)/ps**	**TA (ESA1)/ps**	**literature**(46)**/ps**
1-pentanol	<IRF	9.8 ± 1.8	8.1 ± 1.2	21.7
	250 ± 180	136 ± 37	160 ± 24	151
methanol	<IRF	13.0 ± 1.8	17.9 ± 1.1	15.3
dimethyl sulfoxide	<IRF	5.2 ± 0.3	6.3 ± 1.0	2.29, 10.7

a Note solvation time scales <2 ps were omitted from our analysis. Literature solvent reorganization multiexponential fit parameters for Coumarin 153 were determined by fluorescence upconversion spectroscopy.^46^

The spectral evolution of the transient absorption data were examined to extract key dynamical insights about the excited state evolution of Nile Red. The time-dependent shift of the SE was fitted to a rise followed by a biexponential decay. The rise component is primarily attributed to the feature moving away from a spectrally congested region upon Stokes shifting. The nanosecond SE decay component has a time constant of 3.68 ± 0.18 ns, in good agreement with fluorescence lifetime determined by WR-TCSPC and prior reports (see summary in Table 2). The origin of the second and faster hundreds of picoseconds decay component is discussed later. The evolution of the SE feature was very similar in all the other solvents studied (see the SI for TA data in DMSO, dichloromethane (DCM), methanol, and toluene); however, as expected, minimal red-shifting was observed in less polar solvents such as DCM and toluene (Figures S25 and S28). Further, the time scale for spectral shifting was determined to be in good agreement with the reported excited-state solvation time scales^46^—see Table 1. Simultaneous to the SE red-shift, ESA2 blue-shifts, but ESA1 does not exhibit the same behavior. Further, it is important to note that the time scale of the ESA2 blue-shift is very similar to the observed SE Stokes shift (see Table 1). After vibrational cooling is complete, ESA2 decays biexponentially with time constants of 185 ± 48 ps and 3.87 ± 0.09 ns in 1-pentanol, which are well within the error of the time constants extracted for the SE feature in the TA data. Given the different dynamics of ESA1 and ESA2, we surmise that these two different ESAs have different vertical Franck–Condon (FC) factors from the S_1_ state to the higher-energy excited states, e.g., S_*n*_ ← S_1_ and S_*m*_ ← S_1_ for ESA1 and ESA2, respectively. The lack of observed spectral shifting for ESA1 leads us to suggest that the manifold of S_*n*_ states have similar solvent relaxed minima to S_1_, and thus as S_1_ relaxes, the transition energy does not change. Whereas, S_*m*_ states likely have different minima to S_1_, and so as the S_1_ state relaxes, the FC factors to the S_*m*_ states change, yielding the observed time-dependent spectral shift.

**Table 2:** Summary of the Time Constants for the Different Species Retrieved from WR-TCSPC, TA, and TRIR Compared to Literature Values^a^

**solvent**	**WR-TCPSC/ns**	**TA (ESA2)/ns**	**TA (SE)/ns**	**TRIR (GSB)/ns**	**lit./ns**
1-pentanol		0.185 ± 0.048	0.120 ± 0.019	0.192 ± 0.027	
	3.90 ± 0.18	3.87 ± 0.09	3.95 ± 0.05	3.58 ± 0.34	3.95^51^
DMSO		0.292 ± 0.049	0.292 ± 0.063	0.273 ± 0.038	
	4.37 ± 0.20	4.23 ± 0.13	4.28 ± 0.08	4.44 ± 0.21	4.12^42^
DCM		b0.0089 ± 0.0023	b0.010 ± 0.007	0.059 ± 0.044	
	4.65 ± 0.17	4.01 ± 0.08	4.31 ± 0.06	3.92 ± 0.10	4.48^51^
toluene		0.085 ± 0.0028	0.085 ± 0.0028		
	3.90 ± 0.25	3.76 ± 0.088	3.52 ± 0.14		
methanol		0.013 ± 0.001	b0.017 ± 0.002		
	2.95 ± 0.18	2.87 ± 0.055	3.04 ± 0.05		2.80^51^

a The nanosecond time constant corresponds to the Nile Red fluorescence lifetime; tens to hundreds of picosecond time constants correspond to internal conversion back to S_0_.

b Denotes an exponential rise component arising from spectral shifting away from congested parts of transient spectra.

TRIR spectroscopy was also used to investigate the excited-state dynamics of Nile Red in solvents with some spectral transparency in the mid-infrared. These data contain a strong and isolated vibrational bleach feature at ∼1115 cm^–1^ in DCM-*d*_2_ and 1-pentanol and ∼1580 cm^–1^ for DMSO-*d*_6_—see Figures S29–S31. DFT/ωB97XD/def2-TZVPP calculations assign these vibrational wavenumbers to normal modes dominantly associated with in-plane symmetric ring breathing of the benzo-fused cyclic ketone rings and symmetric ring-breathing of the oxazine ring, respectively. The time-evolution of this bleach feature provides insight into the time scale that molecules repopulate the electronic ground state. The GSB recovery is biphasic in 1-pentanol (see Figure S32), decaying with a 192 ± 27 ps component and a secondary 3.58 ± 0.34 ns time constant. These time constants are in excellent agreement with those extracted from ESA and SE features in TA and WR-TCSPC measurements (Table 2 and Table S3). These observations are repeated in the two other solvents, DCM-*d*_2_ and DMSO-*d*_6_, studied with TRIR—the main time constants are collated in Table 2. DCM-*d*_2_ TRIR data exemplify the kinetics observed in all three TRIR data sets: the prominent and isolated positive features in TRIR data exhibited the same biexponential decay kinetics (Figure S33) as the GSB recovery (Figure S34). Given the match in S_1_ decay and recovery of the S_0_ state for time delays shorter than hundreds of picoseconds, we conclude that, at early time delays, there is a competitive *direct* S_1_ → S_0_ nonradiative pathway that deactivates the S_1_ fluorescent state of Nile Red. A previous study by Cser et al. found a direct correlation between the fluorescence quantum yield and the lifetime of Nile Red. They inferred from their fluorescence measurements that a competitive nonradiative pathway, internal conversion to the ground electronic state, was enhanced by increased solvent hydrogen-bonding interactions with Nile Red. Our transient data broadly reproduce this trend: we find that the amplitude associated with internal conversion (Table S3) increases as a function of the solvent’s hydrogen-bond acidity parameter, Σα_2_^H^,^50^ as shown in Figure S36. Further, as no new positive features appear within the 10 ns time delay investigated, significant intersystem crossing and other photoproduct generating pathways are ruled out for Nile Red after photoexcitation of the S_1_ state.

## 3 Conclusions

Through a combined experimental and theoretical study, we have definitively shown that Nile Red fluorescence originates from a PICT state and *not* a TICT state.^22,23,28−32^ Further, we conclusively refute prior hypotheses that the molecule exhibits dual fluorescence.^20,21,28^ WR-TCSPC and TA data, along with corresponding theory presented here, demonstrate that the early time dynamics of Nile Red are dominated by a dynamic Stokes shift and not interconversion between two excited electronic states as previously claimed. The near-limiting initial value of the fluorescence anisotropy, the lack of any wavelength dependence, and LR-TDDFT calculations further reinforce the conclusion that emission occurs from the same state populated with visible photoexcitation. The dye’s strong solvatochromism has previously been attributed to an emissive excited state with a significant charge-transfer character that will induce a significant solvent reorganization, and some studies concluded that the emissive state is twisted ICT in nature. However, our theoretical results show that the S_1_ state is instead a *planar* ICT state, in agreement with a prior CAM-B3LYP study,^18^ and the LIIC pathway connecting the vertical Franck–Condon region to the S_1_ minimum involves no twisting of the diethylamino group. This is another key result of our study, especially as Nile Red is frequently cited as an archetypical TICT molecule.

Our study underscores, that much like DMABN,^24,26,52^ the importance of combined experimental and theoretical studies to ensure that photochemical dynamics are rigorously interpreted from multiple perspectives, thereby mitigating the risk of misinterpretation. Our comprehensive study of Nile Red has definitively proven that the molecule’s fluorescence originates from a PICT state, despite prior studies classifying Nile Red as an archetype TICT molecule.^22,23,28−32^ In light of our findings, we expect that other molecules that have been proposed to involve TICT states, many of which are used in molecular sensing, will now require careful re-evaluation to establish the physical origin of their environmental sensitivity.

## Data Availability

The data underlying this study are openly available at Zenedo at https:/zenedo.org/doi/10.5281/zenodo.13940659.

## Supporting Information Available

Experimental WR-TCSPC, TA, and TRIR methods; data analysis; fluorescence anisotropy; TRIR and additional TA data; full computational methods and supporting theoretical results including LIIC plots for multiple excited states; and benchmarking (PDF)
