# Supplementary material for: Nile Red Fluorescence: Where’s the Twist?
Source: J Phys Chem B. 2024 Nov 14;128(47):11768–75. doi: 10.1021/acs.jpcb.4c06048 (PMC11613612; doi:10.1021/acs.jpcb.4c06048)
Supplement: Supplementary file 1 — jp4c06048_si_001.pdf [file jp4c06048_si_001.pdf]

Supporting Information for:

Nile Red Fluorescence: Where's the Twist?

Camilla Gajo,[1] Darya Shchepanovska,[1♦] Jacob F. Jones,[1] Gabriel Karras,[2‡] Partha Malakar,[2] Gregory M. Greetham,[2] Olivia A. Hawkins,[1] Caleb J. C. Jordan,[1] Basile F. E. Curchod[1]* and Thomas A. A. Oliver[1]*

[1] School of Chemistry, Cantock's Close, University of Bristol, BS8 1TS, UK

[2] Central Laser Facility, Science and Technology Facilities Council, Research Complex at Harwell, Rutherford Appleton Laboratory, Didcot, Oxfordshire, OX11 0QX, UK

[‡] Current address: Diamond Light Source, Harwell Science and Innovation Campus, Didcot, Oxfordshire, OX11 0DE, UK

[♦] Current address: Met Office, Fitzroy Road, Exeter, EX1 3PB, UK

Authors for correspondence:*basile.curchod@bristol.ac.uk and *tom.oliver@bristol.ac.uk

1. Sample Preparation and Steady State Spectroscopy

Nile Red (99%) was purchased from ThermoFisher Scientific and used without further purification. Nile Red was dissolved in multiple solvents all purchased from Sigma Aldrich (≥99% purity). The steady state absorption was measured on an Agilent Cary UV-visible spectrophotometer and the steady state fluorescence was measured with an Edinburgh instruments FS5 fluorimeter. A low-temperature glass of Nile Red in ethanol at 77 K was generated with an Oxford Instruments Cryostat (Optistat DN). For wavelength-resolved time-correlated single photon counting (WR-TCSPC) measurements, the solutions were diluted to an absorbance of ~0.3 at the peak wavelength of the excitation laser in a static 1 cm pathlength cuvette. For time-resolved infrared (TRIR) measurements, samples were flowed in a Harrick cell of 100 μm pathlength, and diluted to ensure the peak S_1 absorbance was ~0.3. Due to strong solvent absorption of 1-pentanol in the mid-infrared region, the pathlength was reduced to 50 μm, but the absorption maintained at ~0.3 at the pump excitation wavelength. Samples were flowed through a Starna cell (200 μm pathlength) for transient absorption (TA) measurements, where the OD at the S_1 maximum was diluted to be ~0.3.

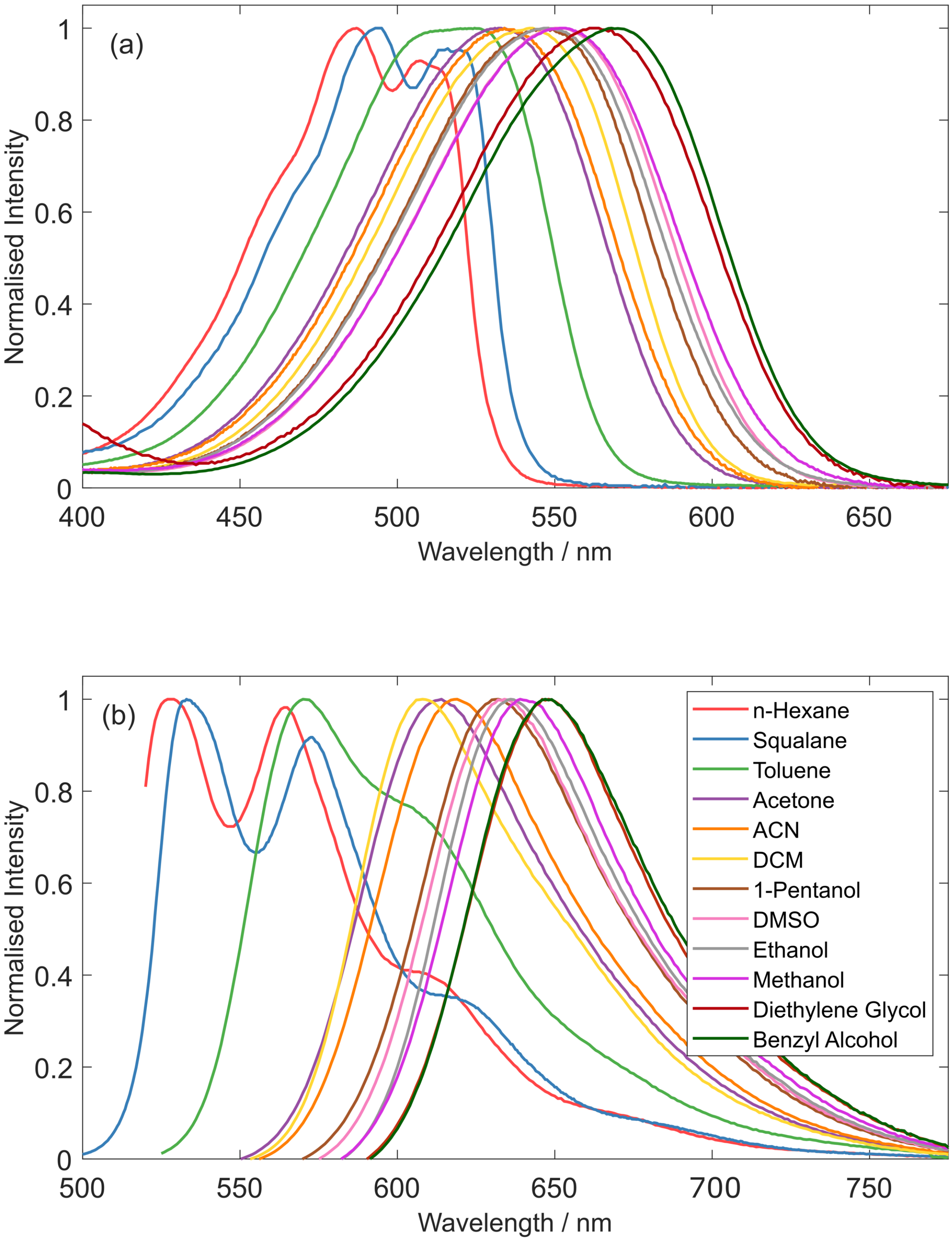

Figure S1. Steady state absorption (a) and fluorescence (b) spectra of Nile Red in 12 solvents. Fluorescence spectra were recorded by exciting samples at their respective absorption maxima using a 1 nm excitation bandwidth.

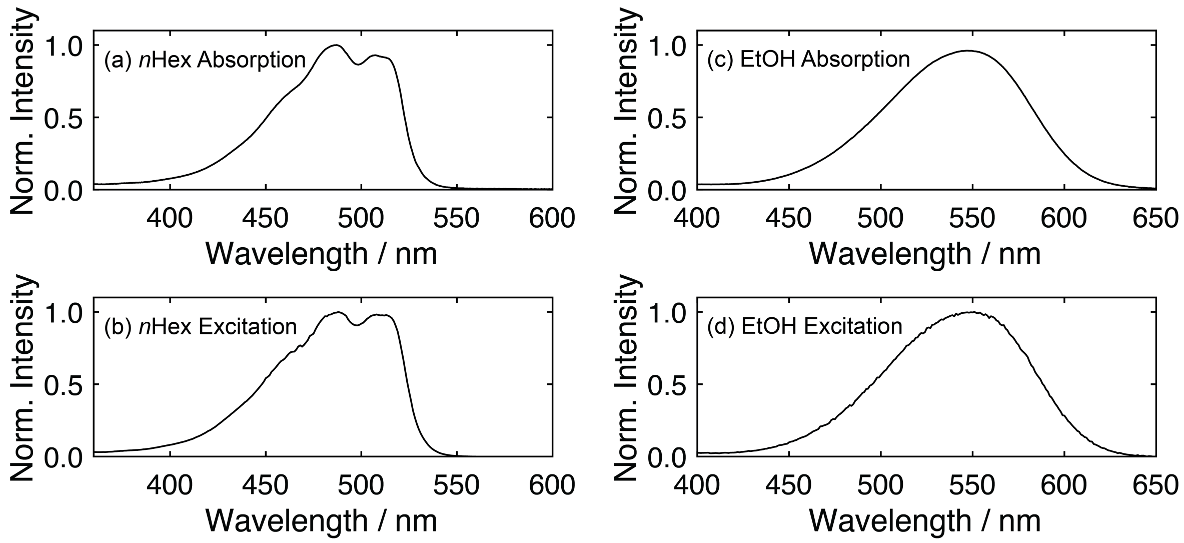

Figure S2. Nile Red steady state absorption and fluorescence excitation spectra in (a,b) *n*-hexane (*n*Hex) and (c,d) ethanol (EtOH) acquired at 293 K. Fluorescence excitation spectra were acquired monitoring signals at the respective fluorescence maxima.

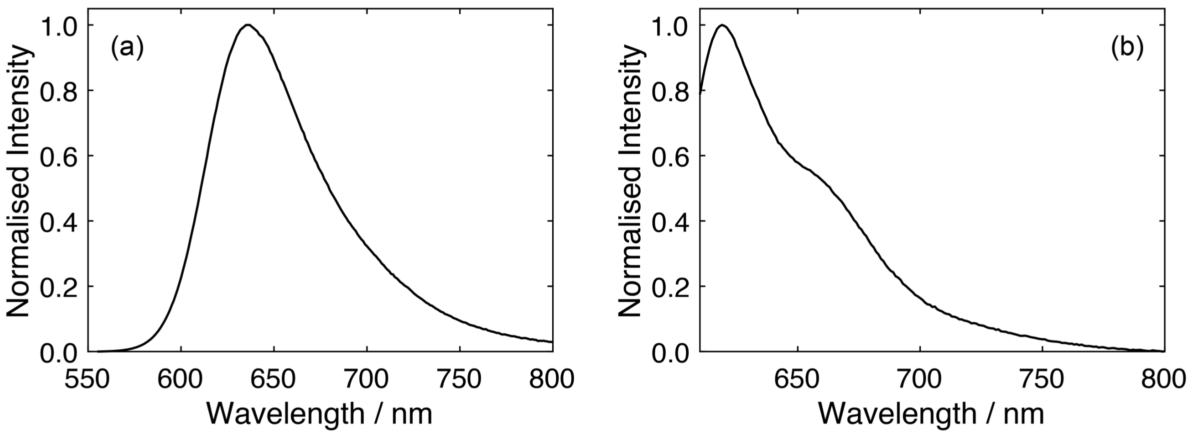

Figure S3. Comparison of Nile Red fluorescence in ethanol at (a) 293 K and (b) 77 K after 550 nm excitation.

Table S1. Absorption and fluorescence maxima of Nile Red in 12 different solvents. These maxima either corresponded to the maximum intensity, or where data contained vibrational structure, the maximum of the first vibrational band was chosen.

Solvent	Abs. Max. / nm	Fluo. Max. / nm
n-Hexane	508 ± 4	528 ± 2
Squalane	518 ± 3	534 ± 2
Toluene	522 ± 4	571 ± 2
Acetone	532 ± 2	613 ± 2
Acetonitrile (ACN)	535 ± 3	619 ± 2
Dichloromethane (DCM)	542 ± 2	608 ± 1
1-Pentanol	546 ± 3	632 ± 3
Dimethyl sulphoxide (DMSO)	551 ± 2	634 ± 2
Ethanol	547 ± 1	634 ± 2
Methanol	553 ±1	639 ± 2
Diethylene Glycol	562 ± 2	648 ± 2
Benzyl Alcohol	570 ± 2	648 ± 2

2. Experimental Methods

2.1 Wavelength-Resolved Time-Correlated Single Photon Counting

WR-TCSPC measurements were acquired using a previously reported homebuilt apparatus.[1] Briefly, the output of a tuneable high-power ultrafast oscillator (Chameleon Ultra II, 3.7 W, 80 MHz, Coherent) was used to pump a commercial optical parametric oscillator (Chameleon Compact OPO, Coherent/APE) to generate near-IR pulses between 1020 and 1100 nm, which were frequency doubled in a BBO crystal ($\theta = 29.2°$, 2.5 mm Eskma) to generate the required excitation pulses (510–550 nm). To avoid re-excitation of samples, the repetition rate was reduced to 6.67 MHz using a pulse picker (APE cavity dumper). The diffracted output was then focused into a static liquid sample (1 cm pathlength cell) with energies <10 pJ per pulse. Fluorescence was collected at 90º relative to excitation using an infinity-corrected microscope objective (4×/0.2 NA Plan Apochromat, Nikon), and through a series of achromatic lenses collimated and focused onto an avalanche photodiode detector (ID100-50-ULN, IDQ).

Photon arrival times were recorded with a time-to-digital converter (Time Tagger 20, Swabian Instruments) and accumulated in 10 ps bins. To facilitate wavelength-resolved TCSPC measurements, fluorescence was propagated through a birefringent interferometer (Gemini, Nireos)[2] placed before the detector. Each data point was integrated for 0.5 s, and every spectral interferogram was averaged 10 times. All WR-TCSPC data were acquired using customized LabVIEW software (National Instruments). Spectral filtering due to transmission through the collection objective and Gemini interferometer were corrected for in post-processing. The instrument response function (IRF) was ~180 ps, as determined by recording laser scatter from solvent in a 1 cm pathlength cuvette. All data were collected at room temperature (20 °C) using the magic angle condition.

2.2 Transient Absorption and Time-Resolved Spectroscopy

Time-resolved infrared (TRIR) spectroscopy measurements were performed at the ULTRA Central Laser Facility (CLF) using the LIFEtime laser system, and the experimental apparatus has been previously reported.[3,4] Briefly, this is a synchronised dual amplifier-based multiple probe spectroscopy setup, driven by two 1030 nm, 100 kHz, 15 W Yb:KGB laser amplifiers (Pharos, Light Conversion Ltd.). Tuneable visible pump pulses are produced by an optical parametric amplifier (OPA) at a repetition rate of 1 kHz (reduced by the internal Pharos laser pulse-picker). The mid-infrared probe pulses (between 1080–1670 cm^{-1} each with a central bandwidth of ~150 cm^{-1}) were generated with a commercial OPA at 100 kHz repetition rate. The samples were pumped at wavelengths corresponding to the peak of the S_1 absorption band (542, 546 nm and 557 nm in DCM-d_2, 1-pentanol and DMSO-d_6, respectively) with 450 nJ focused at 120μm diameter spot at the sample position. The transmitted probe and colinear signals were then imaged into a spectrograph, frequency dispersed onto a 128-element HgCdTe mid-IR array detector (Infrared Associates Inc).

Transient absorption (TA) data were recorded at the University of Bristol using a similar synchronised dual amplifier laser system (PH2, Light Conversion). The first amplifier operated at 0.5 kHz and pumped a commercial OPA to generate laser pulses to photoexcite Nile Red. A small fraction of the 1030 nm fundamental from the second amplifier (operating at 1 kHz) was focused into a continually rastered CaF_2 window to generate a white light supercontinuum spanning 350–800 nm. Residual 1030 nm light was removed with a piece of Schott glass. TA spectroscopy investigated Nile Red dissolved in DCM, methanol, DMSO, toluene and 1-pentanol and excited with 542 553, 557, 522 and 546 nm pump light, respectively. These experiments used a pump power of 100 nJ, and focussed to a spot size of 120 μm (diameter, FWHM) at the sample position, and the probe spot size was ~1/2 that of the pump. The probe and signal beams were collimated and imaged into a homebuilt spectrograph coupled to a 1024

CCD camera (Entwicklungsbuero EB Stresing). The instrument response function was determined by non-resonant 2-photon absorption in toluene solvent to be 180 fs. TA data were averaged for 500 shots for each pump-probe time delay, and the time points were randomised. These data comprised one cycle, and for all the TA data reported, the data were averaged for 20 cycles. Raw TA data were chirp-corrected using in-house customised Matlab scripts by fitting the wavelength-dependent instrument response to a polynomial function and shifting all probe wavelengths to a common time zero.

All ultrafast transient data collected at Bristol and the CLF were collected at room temperature (20 °C) and the relative polarisation of the pump and probe laser lines set to magic angle.

3. Computational Details and Additional Computational Results

All calculations presented in the main text were performed with density functional theory (DFT) and linear-response time-dependent density functional theory (LR-TDDFT).[5] The long-range corrected functional ωB97XD [6] was used in combination with a def2-TZVPP [7] basis set for all the atoms. LR-TDDFT employed both the Tamm-Dancoff and the adiabatic approximations. Geometry optimizations were performed *in vacuo* and within a polarizable continuum model (using the integral equation formalism) for each solvent under consideration, and following a search for conformers. We verified the nature of all stationary points located with harmonic vibrational frequency calculations. All static calculations were carried out with the Gaussian 16 package (revision B), and molecular representations were produced with VMD version 1.9.2.[8]

The results obtained with LR-TDDFT/TDA/ωB97XD/def2-TZVPP were further benchmarked (in gas phase) with RI-CC2/def2-SVP[9] using Turbomole 7.4.1.[10] This benchmark was achieved by recalculating the electronic energies with RI-CC2/def2-SVP[9] for each geometry of the LIICs pathways (obtained from the critical geometries obtained with LR-TDDFT/TDA/ωB97XD/def2-TZVPP in gas phase). We tested that the RI-CC2 results are converged with respect to the basis set by performing single-point calculations with def2-TZVPP. Figure S4 shows the overall agreement between the LR-TDDFT/TDA/ωB97XD and RI-CC2 LIIC pathways, in particular for the energy ordering of the minima along the S_1 electronic state. We note that the LIIC pathways presented in Figure S4 exhibit another minimum located on the S_1 electronic state of $n\pi^*$ (the transition to this electronic-state character is clear from the loss of its oscillator strength with S_0 – see the circle sizes in Figure S4 along the S_1 electronic state). In gas phase, this electronic-state character can be found as a minimum on S_1, but its energy is lifted above S_1 as soon as a solvent is implicitly

included (see Figure S5). The structural similarities between the S_0 and S_1(PICT) molecular geometries are highlighted in Figure S6.

Different strategies were deployed to search for a minimum of the first excited electronic state that would exhibit a twisted ICT character, but our searches were unsuccessful using LR-TDDFT/TDA/ωB97XD. A TICT structure could be located with LR-TDDFT/TDA/def2-SVP but using the hybrid functional PBE0, a family of functional known to underestimate the energy of the charge-transfer state of Nile Red and describing an artificial TICT minimum geometry.[11] Any attempt to reoptimise this TICT structure in the first excited electronic state using ωB97XD (with and without the inclusion of the IEFPCM solvation model) switched back its geometry to planar ICT (again, in line with ref. [11]). Nevertheless, this geometry allowed us to probe further the description of the TICT with ωB97XD. We calculated electronic energies along a LIIC pathway connecting the ground-state optimised geometry to the artificial TICT minimum (Figure S7) – highlighting that the electronic energy of the S_1 state (obtained with LR-TDDFT/TDA/def2-TZVPP/ωB97XD) dramatically rises along this twisting coordinate, with and without the inclusion of an implicit solvent for 1-pentanol, in line with ref. [11].

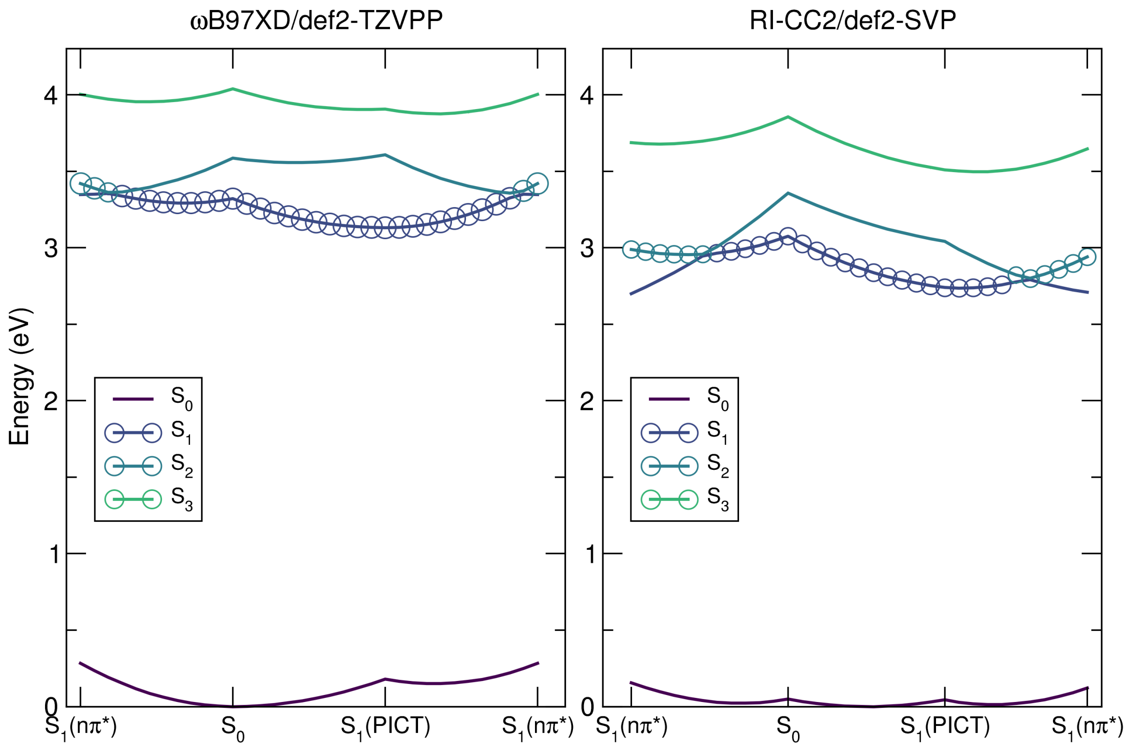

Figure S4. Gas phase LIIC pathways benchmarking the (LR-TD)DFT/TDA/ωB97XD/def2-TZVPP results with RI-CC2/def2-SVP. The LIIC pathways are based on (LR-TD)DFT/TDA/ωB97XD/def2-TZVPP critical geometries. Circle size is proportional to the oscillator strength associated to a given electronic transition.

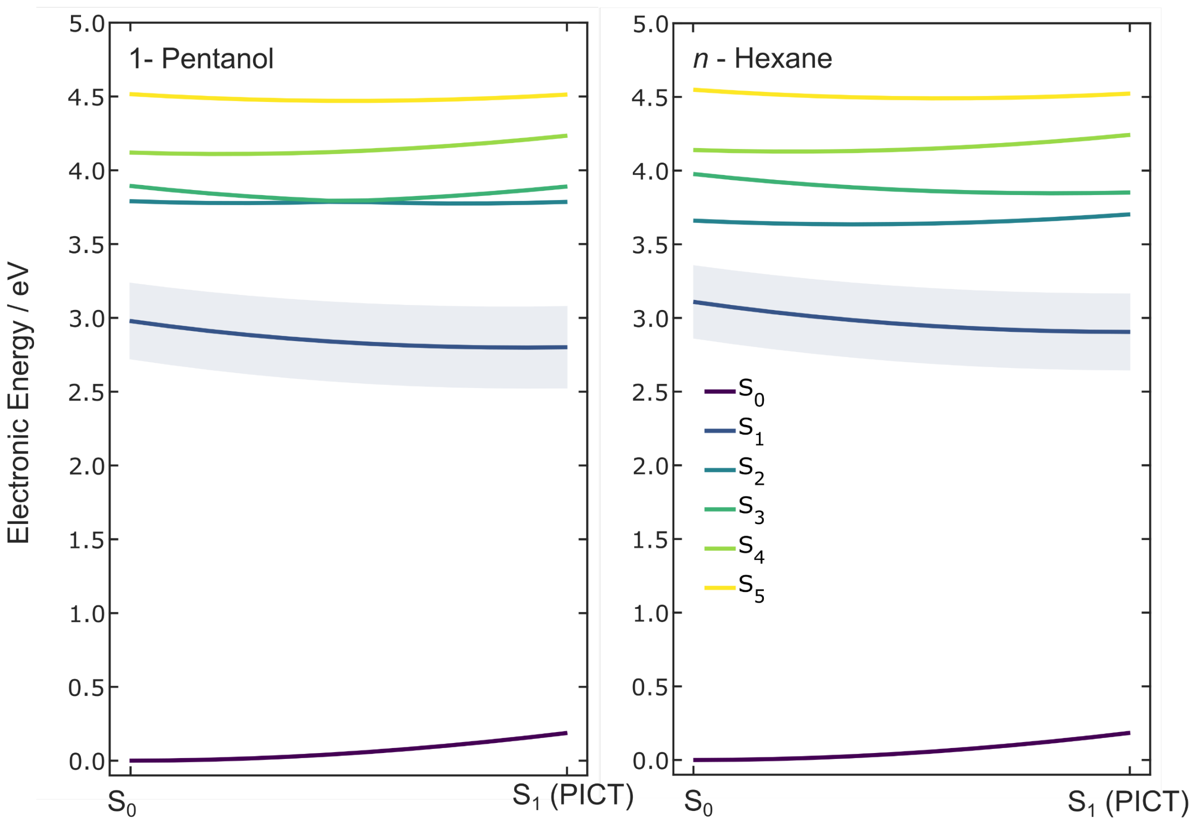

Figure S5. LIIC plot for NR in 1-pentanol for the ground state S_0, and S_1–S_5 excited states.

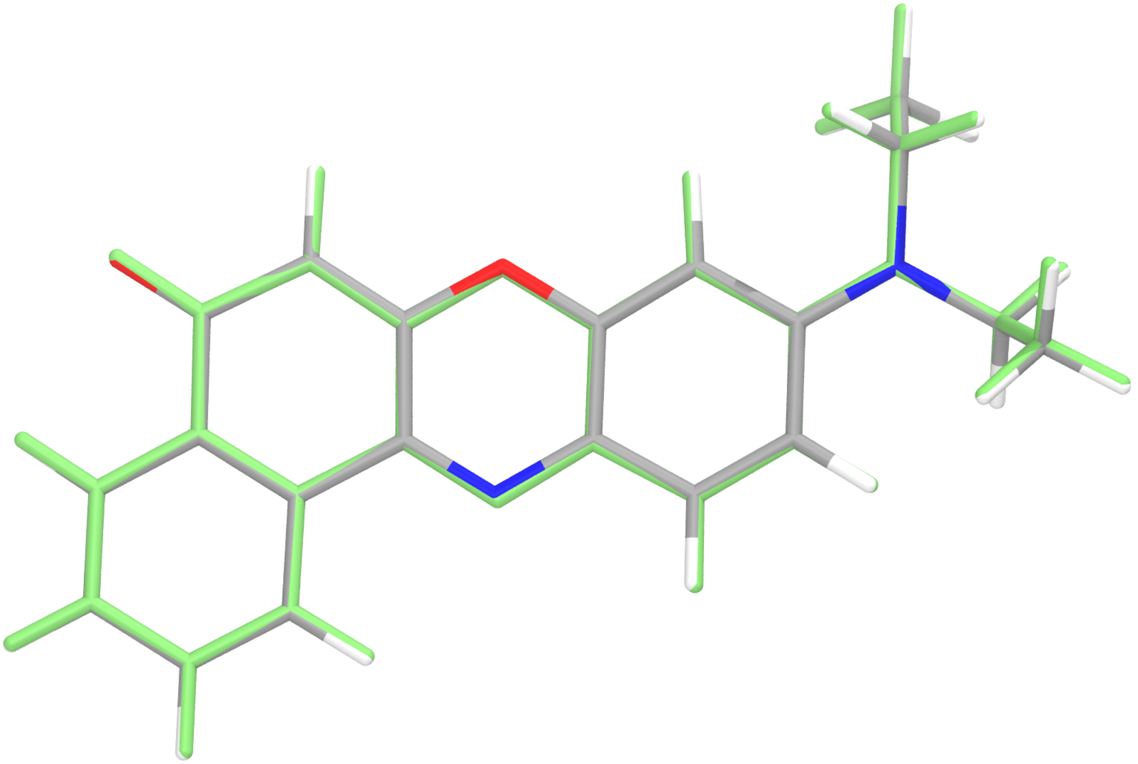

Figure S6. Superimposed optimised S_0 and S_1(PICT) geometries of Nile Red. The S_1(PICT) geometry is depicted in a lime colour.

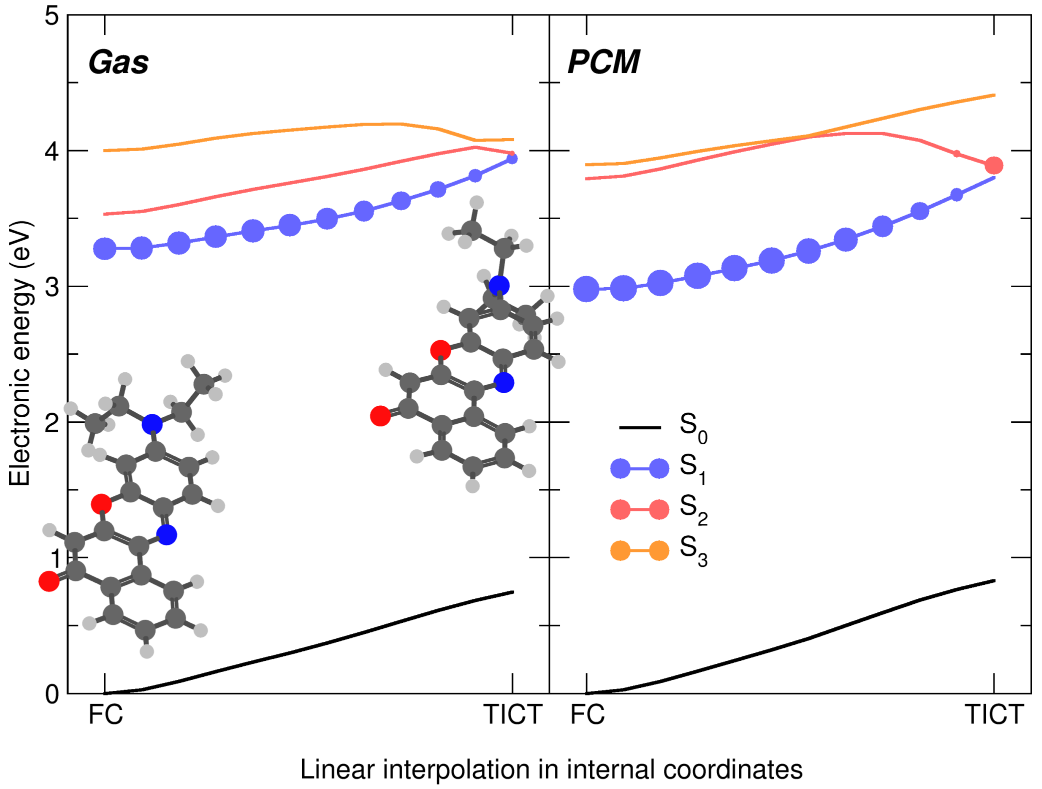

Figure S7. LIIC pathway connecting the S_0 (Franck-Condon) geometry obtained with DFT/ωB97XD/def2-TZVPP to the artificial TICT geometry obtained with LR-TDDFT/TDA/PBE0/def2-SVP (see text). The electronic energies were obtained with (LR-TD)DFT/TDA/ωB97XD/def2-TZVPP, in gas phase (left panel) and with the inclusion of an implicit solvent (IEFPCM) for 1-pentanol.

4. Fluorescence Anisotropy

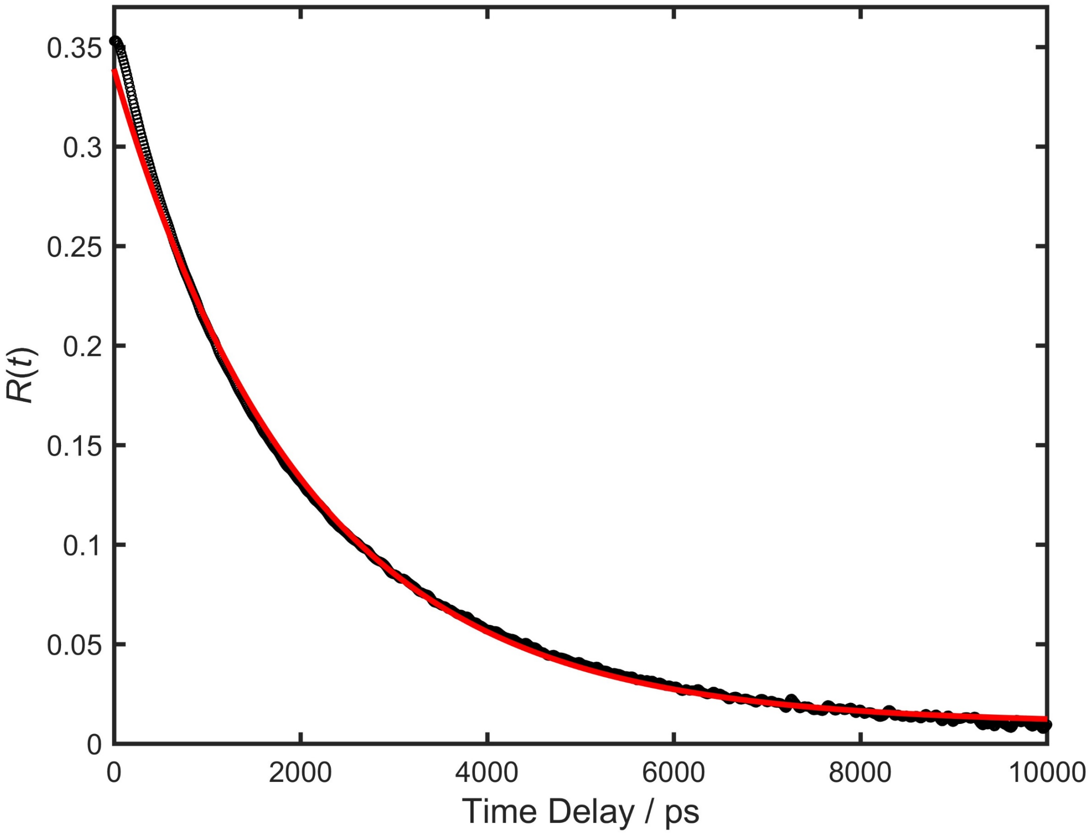

Figure S8. Time-resolved fluorescence anisotropy data taken on the TCSPC setup as described above in highly viscous solvent Squalane. The plot above is a result of 5 averaged sequential measurements taken with an excitation wavelength of 520 nm. The fluorescence was collected with a 550 nm long pass filter and the $R(t = 0\text{ ps})$ was found to be +0.353 ± 0.002.

Fluorescence anisotropy measurements were conducted using the TCSPC apparatus described in section 2.1 but without the commercial GEMINI interferometer. A polariser was placed before the detector, and the electric vector of the excitation laser set with a λ/2 waveplate prior to the sample cuvette. The fluorescence decay traces were recorded at parallel and perpendicular polarisations by rotation of the half-wave plate. From these data, the fluorescence anisotropy was calculated using the following equation:

$$R(t) = \frac{I_{para} - I_{perp}}{I_{para} + 2I_{perp}}. \tag{S1}$$

Where I_{para} and I_{perp} are the measured time-dependent fluorescence intensities at parallel and perpendicular polarisations, respectively. These measurements were conducted in viscous solvent squalane with an excitation wavelength of 520 nm and repeated 5 times. The fluorescence was collected with a 550 nm long pass filter to reduce the amount of scatter being collected. At t = 0 ps the anisotropy value $R(t$ = 0 ps) was determined to be +0.353 ± 0.002 (error calculated from standard deviation of repeats), and close to the limiting value of +0.4, expected in the case of parallel excitation and emission transition dipole moments. The experiment was repeated using 532 nm and a 635 nm bandpass filters instead, that yielded an $R(t$ = 0 ps) of +0.353 ± 0.009 and +0.350 ± 0.002, respectively These values are in good agreement with previously measured $R(t$ = 0 ps) values of + 0.31 ± 0.01 of Nile Red in 1-propanol.

5. Data Analysis

Kinetics at different fluorescence wavelength ranges of WR-TCSPC data were fitted to a multi-exponential decay function:

$$I(t) = A_1 e^{-k_1 t} + A_2 e^{-k_2 t}, \tag{S2}$$

which was convolved with a Heaviside step function and Gaussian instrument response function (IRF). In equation S2, k_1 and k_2 are the associated decay rate constants, where A_1 and A_2 are the associated relative amplitudes. In the cases of fitting a rise component, one of the amplitudes is fixed to have a negative value.

TA data were also fitted with equation S2. TRIR data used the same fitting routine, but the sum of exponentials was not convolved with an IRF because of the (sometimes significant) perturbed free-induction decay at negative time delays. Further, in these TRIR measurements the IRF was considerably shorter than the dynamics of interest.

To extract wavelength shifting dynamics the following procedure was used. Firstly, the region of interest was fitted to a 9th order polynomial to accurately determine the peak. Then, from the fit, the maximum point of each trace was extracted and plotted as shown in Figure S9 and subsequently fitted to an exponential function.

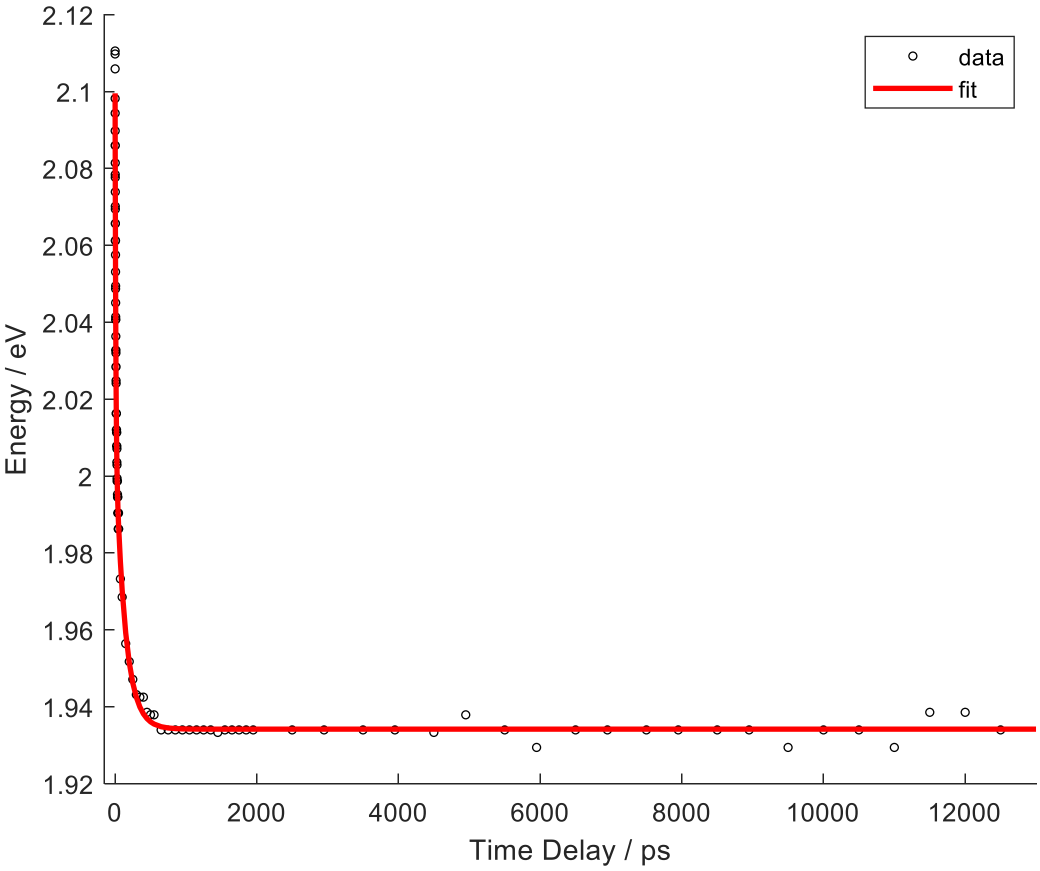

Figure S9. Time dependent shift of SE of Nile Red in 1-pentanol (open circles), with overlaid fit to data (red solid line).

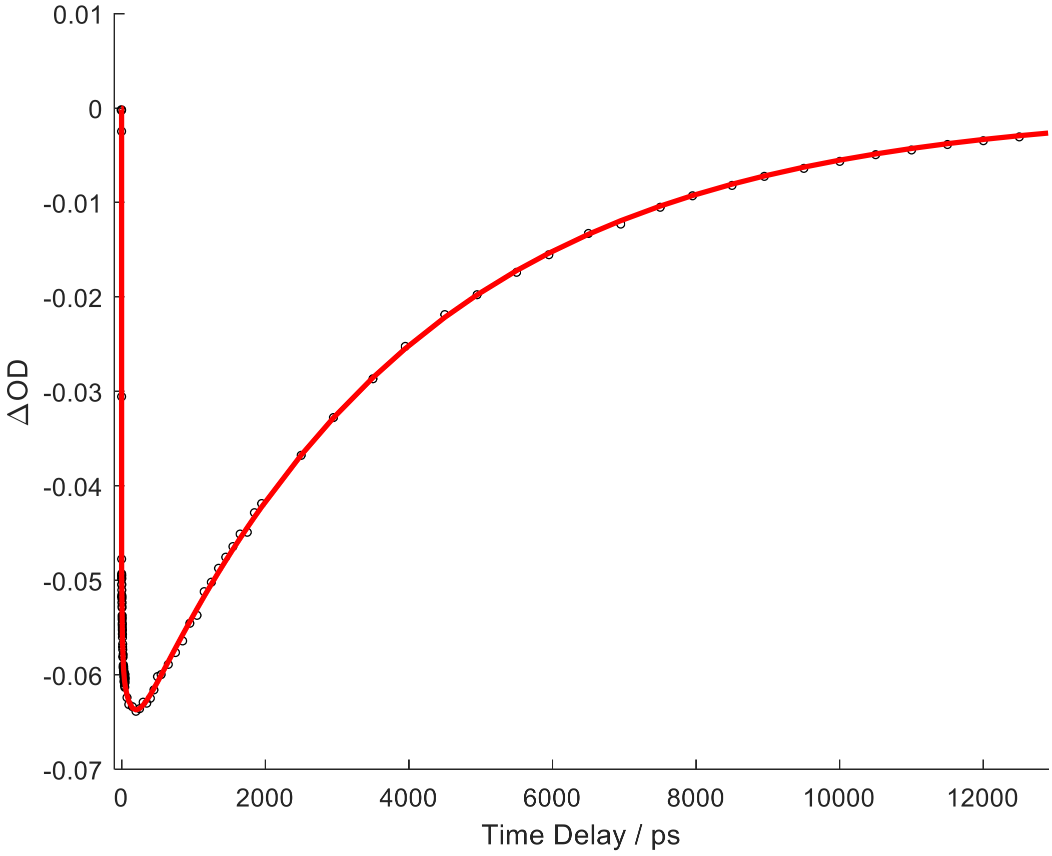

Figure S10. Kinetics associated with the decay of SE of Nile Red in 1-pentanol (open circles), with overlaid fit to data (red solid line).

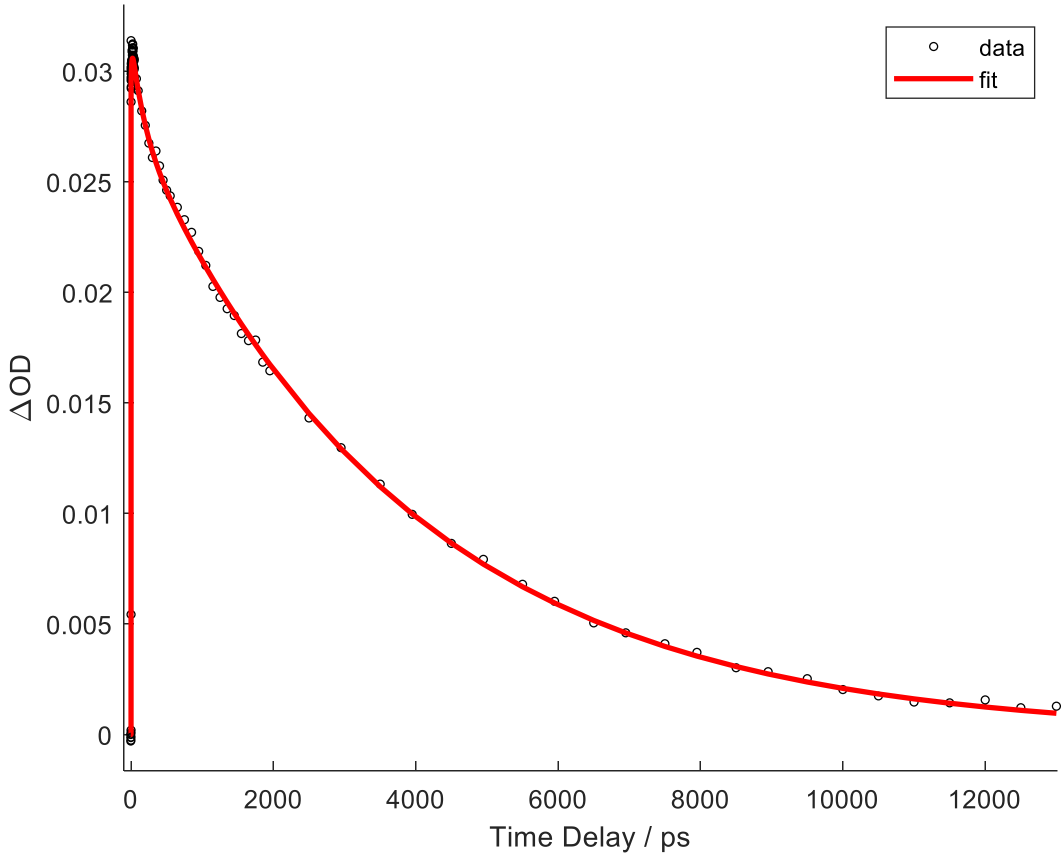

Figure S11. Kinetics associated with the maximum of ESA2 of Nile Red in 1-pentanol (open circles), with overlaid fit to data (red solid line).

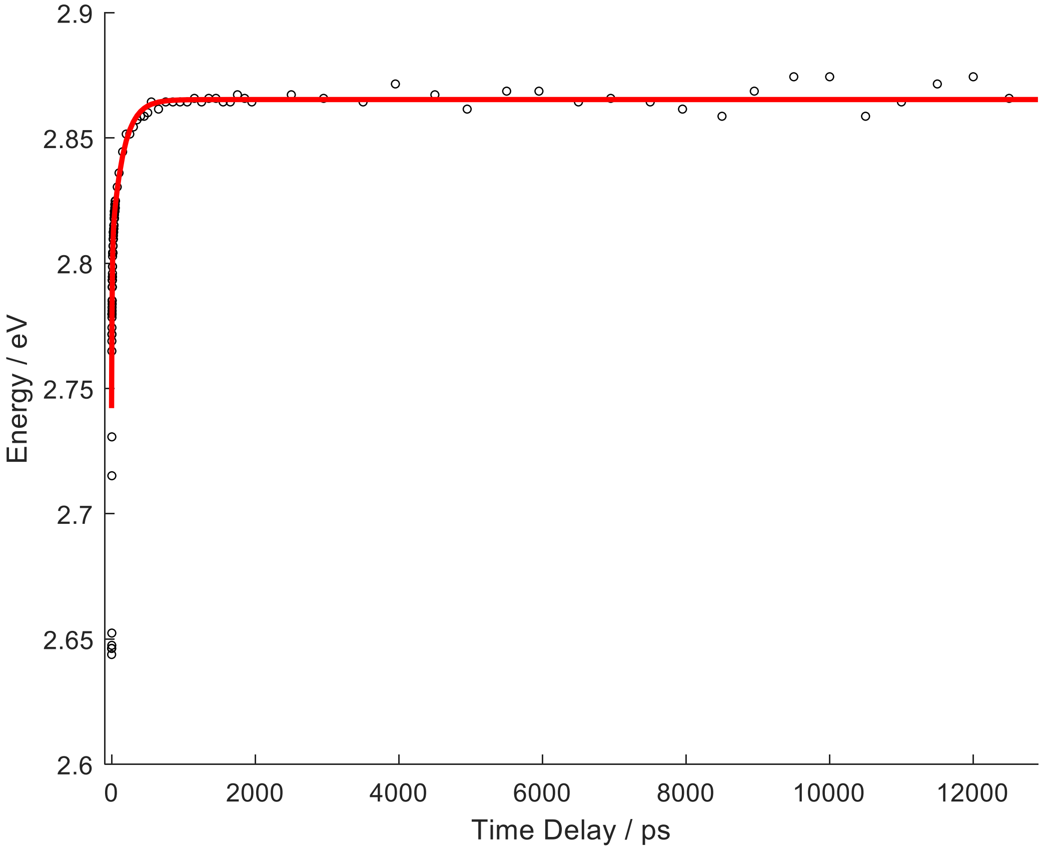

Figure S12. Time dependent shift of ESA2 of Nile Red in 1-pentanol (open circles), with overlaid fit to data (red solid line).

6. Wavelength-Resolved Time-Correlated Single Photon Counting Data

WR-TCPSC data for Nile Red was collected in 12 different solvents (see Table S1 for a list). All solvents (except for very viscous and polar solvents, 1-pentanol, benzyl alcohol and diethylene glycol) showed no dynamic Stokes shift as exemplified by the contour map of *n*-hexane shown in Figure S13 and the extracted kinetics at several different wavelengths shown in Figure S14. At the investigated wavelengths, the extracted timescales of fluorescence are the same (within error). Only when investigating high viscosity and polar solvents such as 1-pentanol, diethylene glycol and benzyl alcohol was a dynamic Stokes shift observed as evident from the contour map of diethylene glycol in Figure S24. High viscosity, low polarity solvents such as squalane displayed no wavelength dependent kinetics – see Figure S22.

Table S2. Nanosecond fluorescence lifetimes of Nile Red extracted from WR-TCSPC data.

Solvent	$\frac{\varepsilon - 1}{\varepsilon + 1} + \frac{n^2 - 1}{n^2 + 2}$	Viscosity at 293 K / mPa·S [12]	$E_T(30)$ kcal mol^{-1} [12]	τ_f / ns
Squalane	–0.0368	31.12	—	3.41 ± 0.17
n-Hexane	–0.0021	0.31	30.9	2.62 ± 0.17
Toluene	0.0244	0.59	33.9	3.90 ± 0.25
Acetone	0.6503	0.32	42.2	4.70 ± 0.20
ACN	0.7125	0.37	45.6	4.67 ± 0.20
Methanol	0.7121	0.60	55.5	2.95 ± 0.18
Ethanol	0.6699	1.20	51.9	3.68 ± 0.18
1-Pentanol	0.5799	3.51	49.1	3.90 ± 0.18
DCM	0.4764	0.44	41.1	4.65 ± 0.17
DMSO	0.6578	2.24	45.0	4.37 ± 0.20
Diethylene Glycol	0.6437	30.00	53.8	3.54 ± 0.17
Benzyl Alcohol	0.4864	5.47	50.8	3.74 ± 0.17

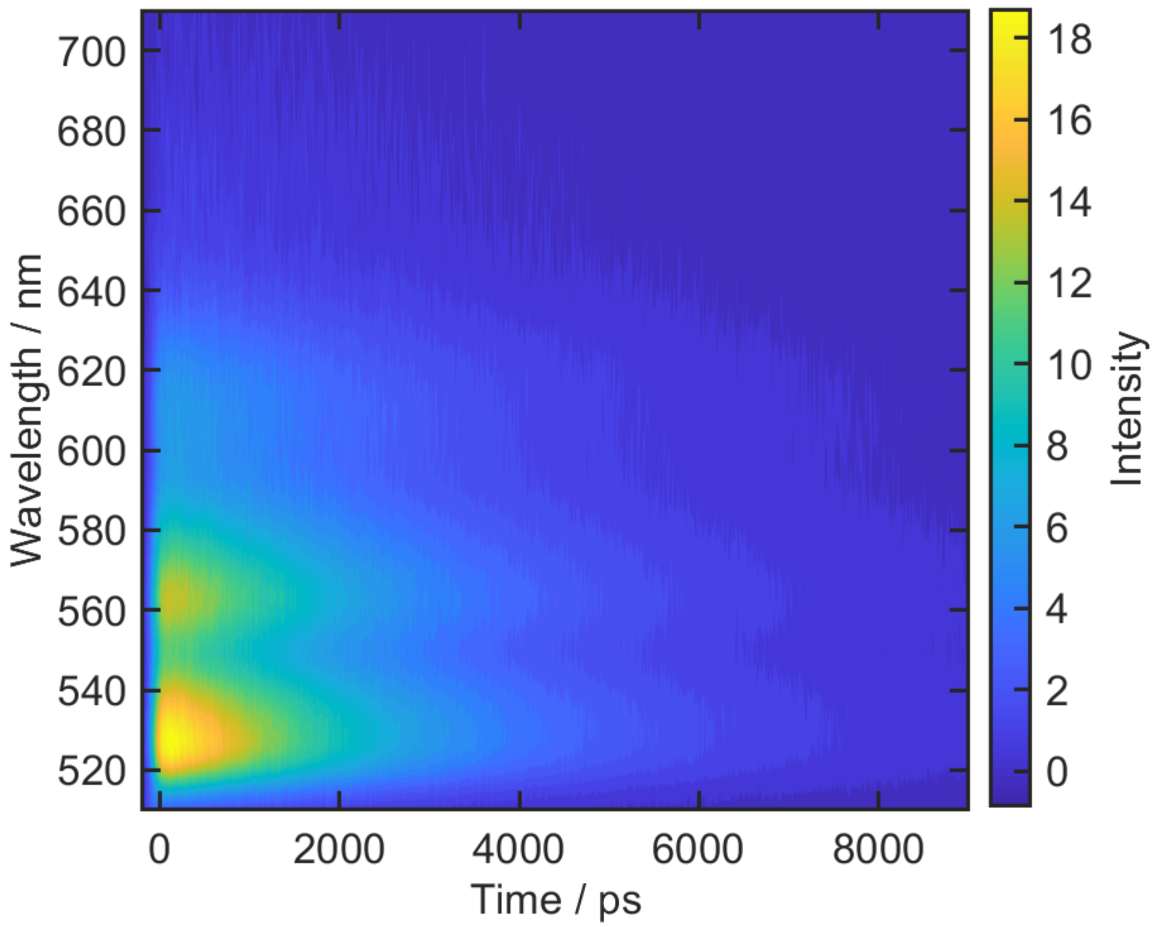

Figure S13. WR-TCSPC contour map of Nile Red in *n*-hexane using 510 nm photoexcitation.

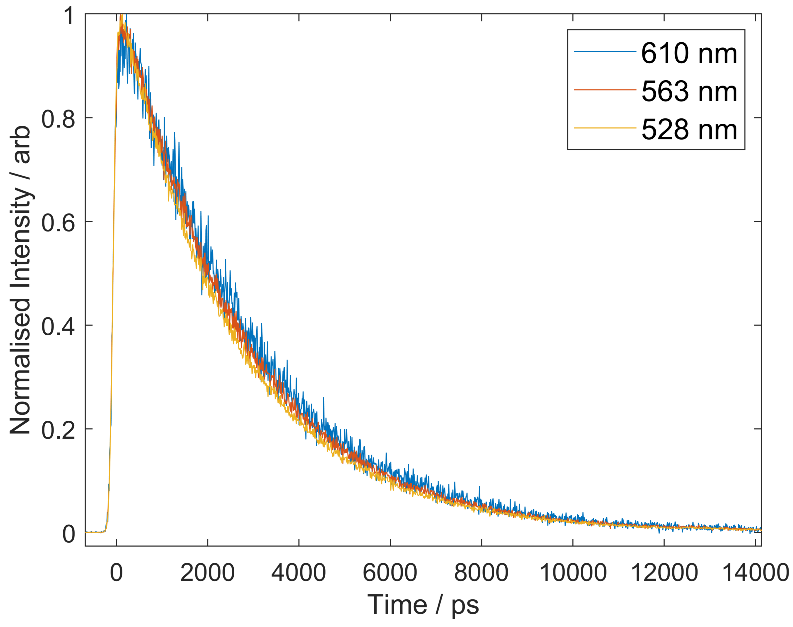

Figure S14. Kinetics for three different fluorescence wavelengths extracted from data displayed in Figure S13.

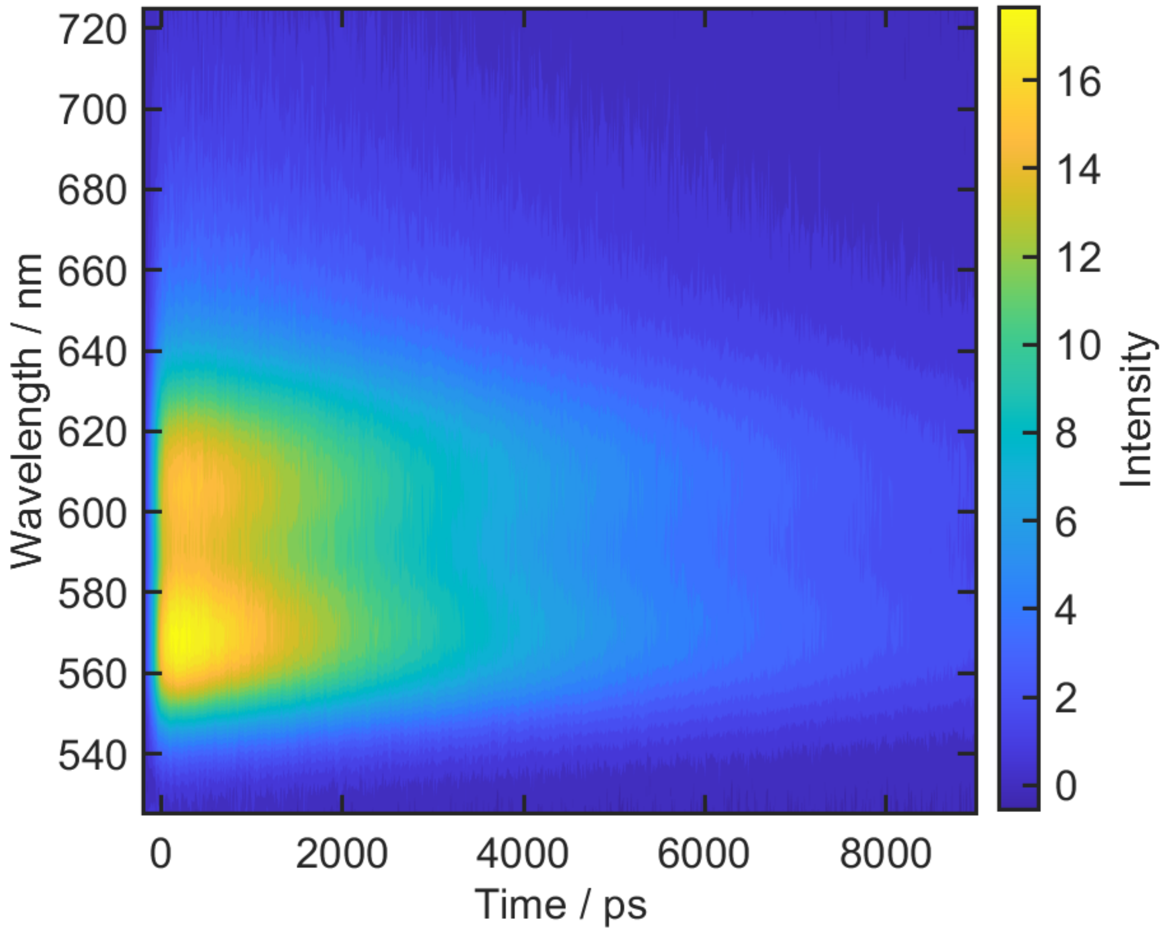

Figure S15. WR-TCSPC contour map of Nile Red in toluene using 520 nm photoexcitation.

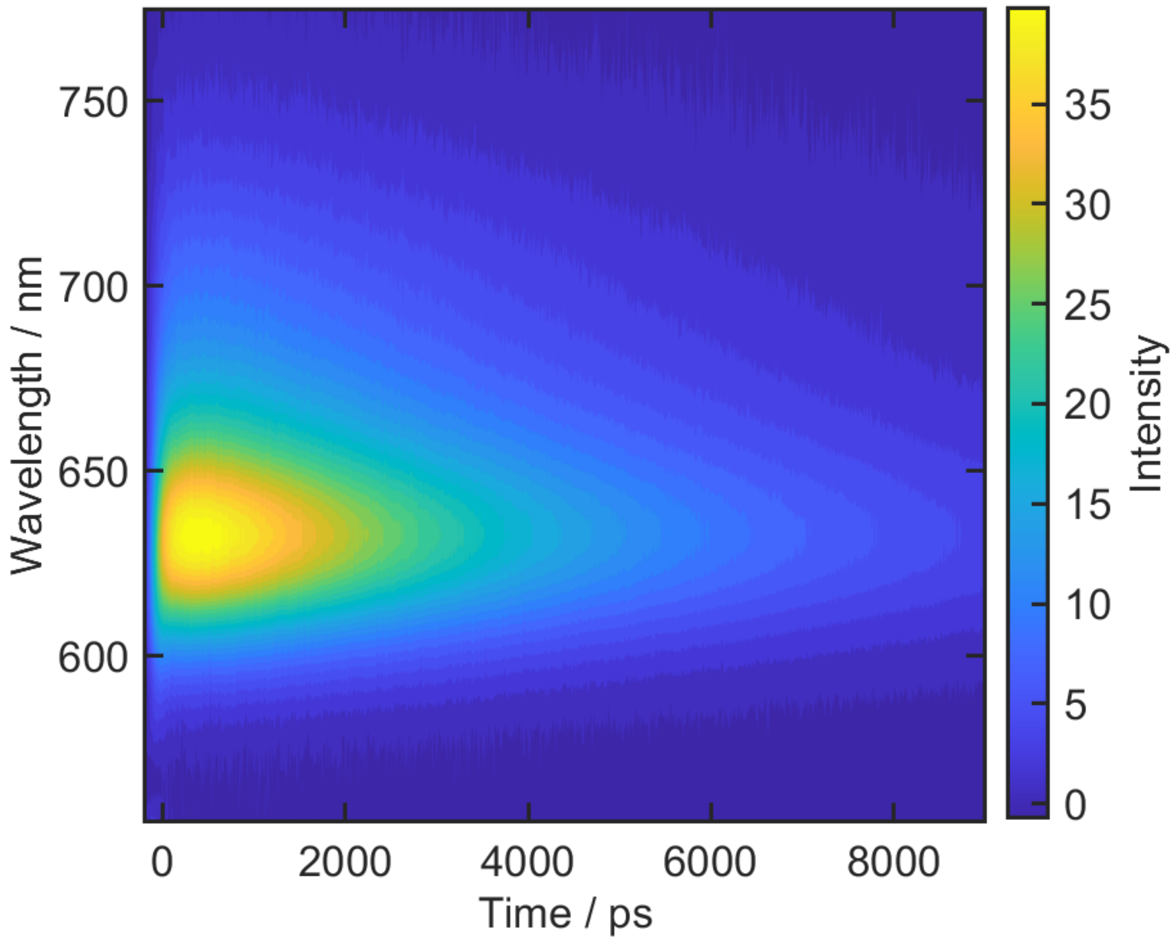

Figure S16. WR-TCSPC contour map of Nile Red in ethanol using 550 nm photoexcitation.

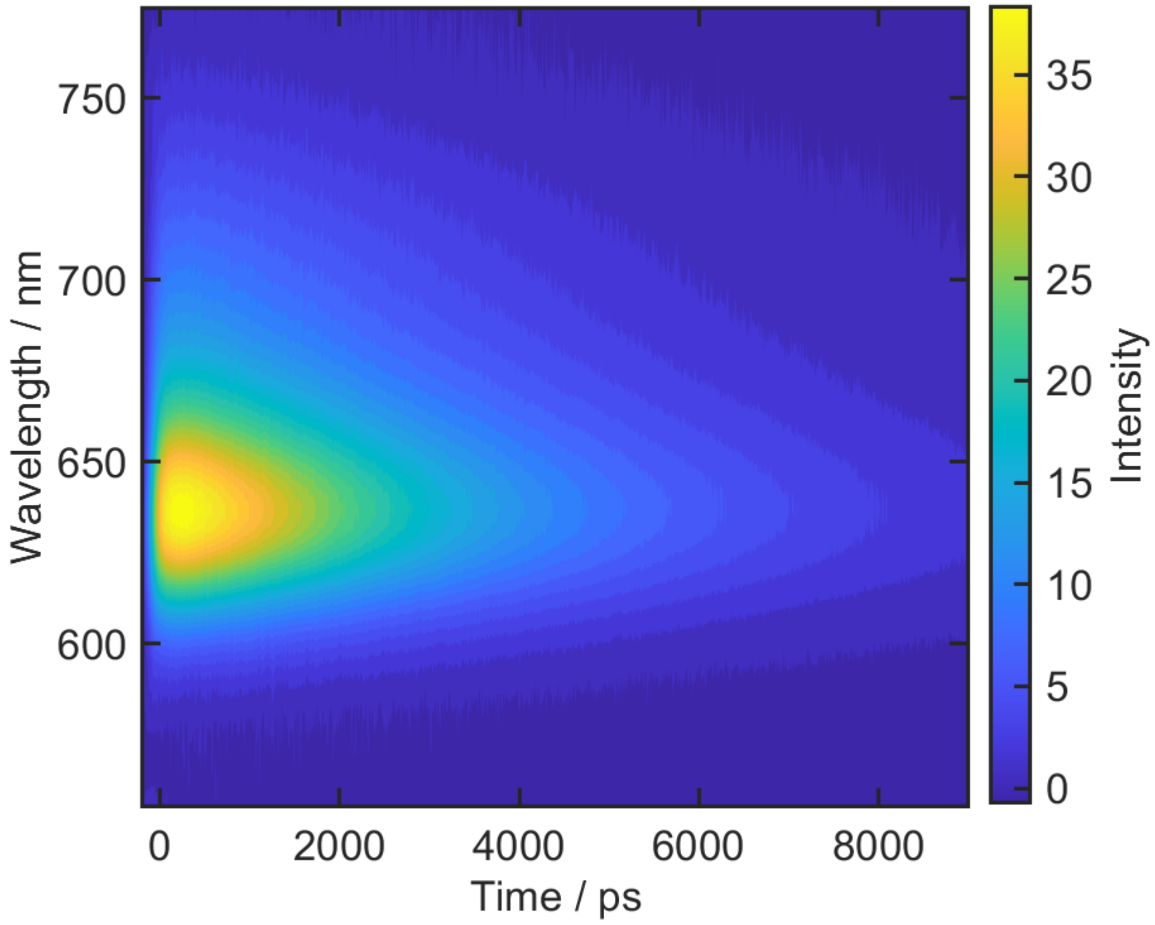

Figure S17. WR-TCSPC contour map of Nile Red in methanol using 550 nm photoexcitation.

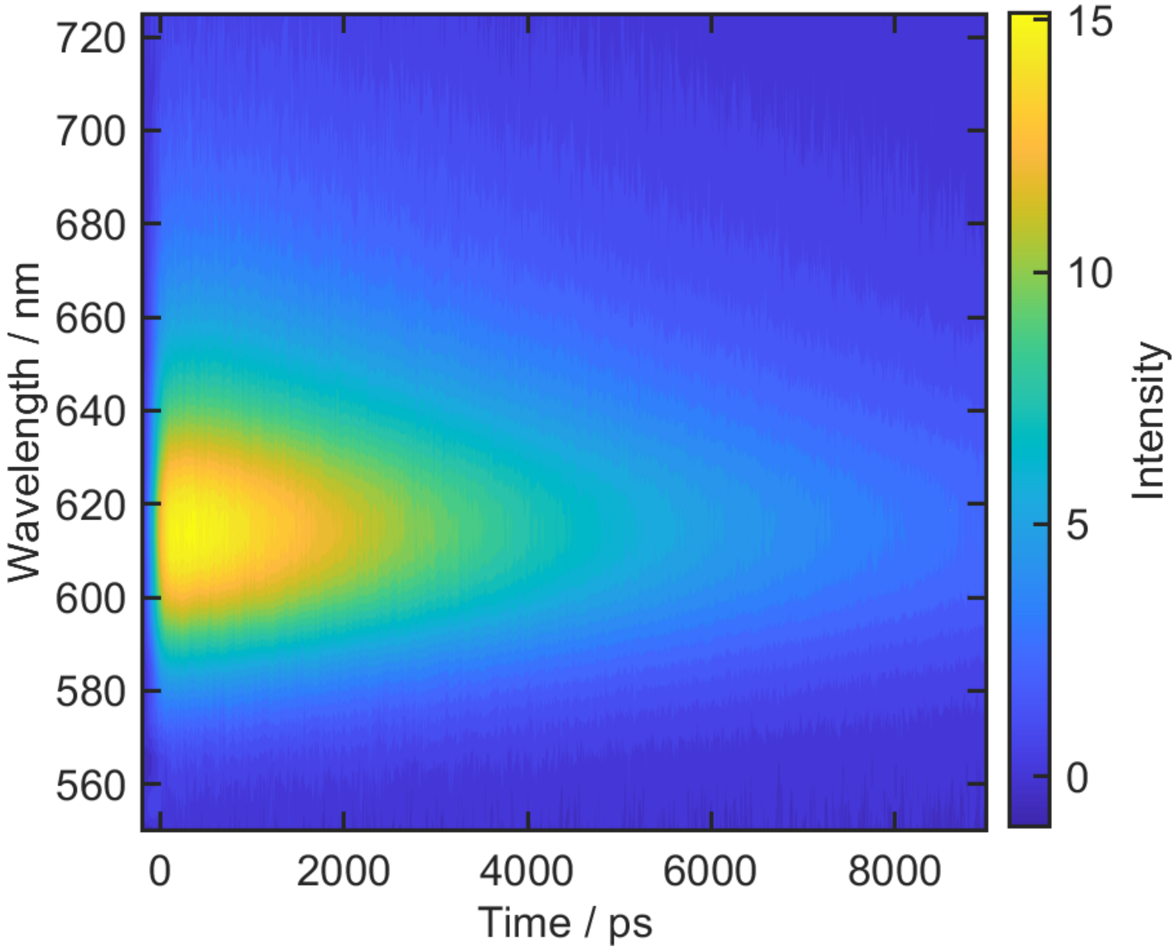

Figure S18. WR-TCSPC contour map of Nile Red in acetone using 534 nm photoexcitation.

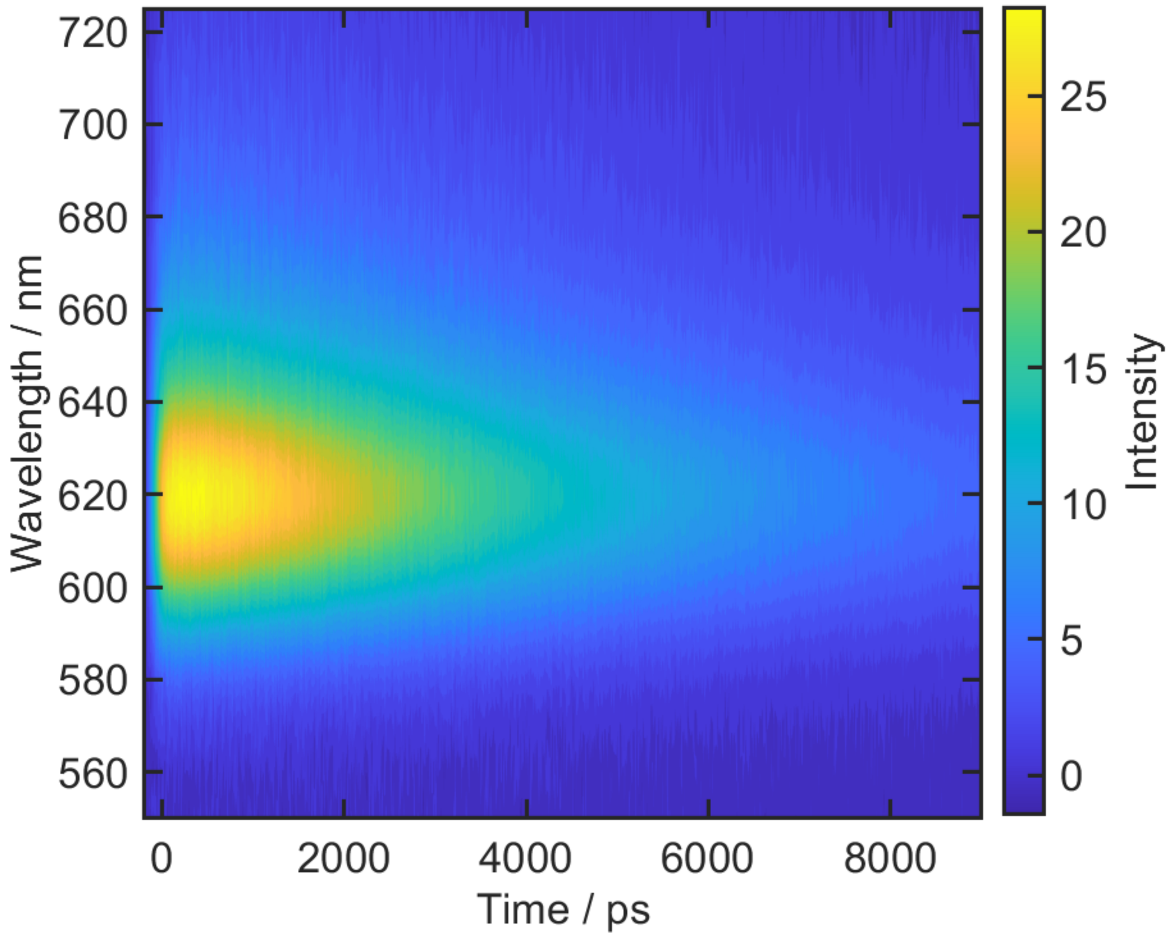

Figure S19. WR-TCSPC contour map of Nile Red in acetonitrile using 534 nm photoexcitation.

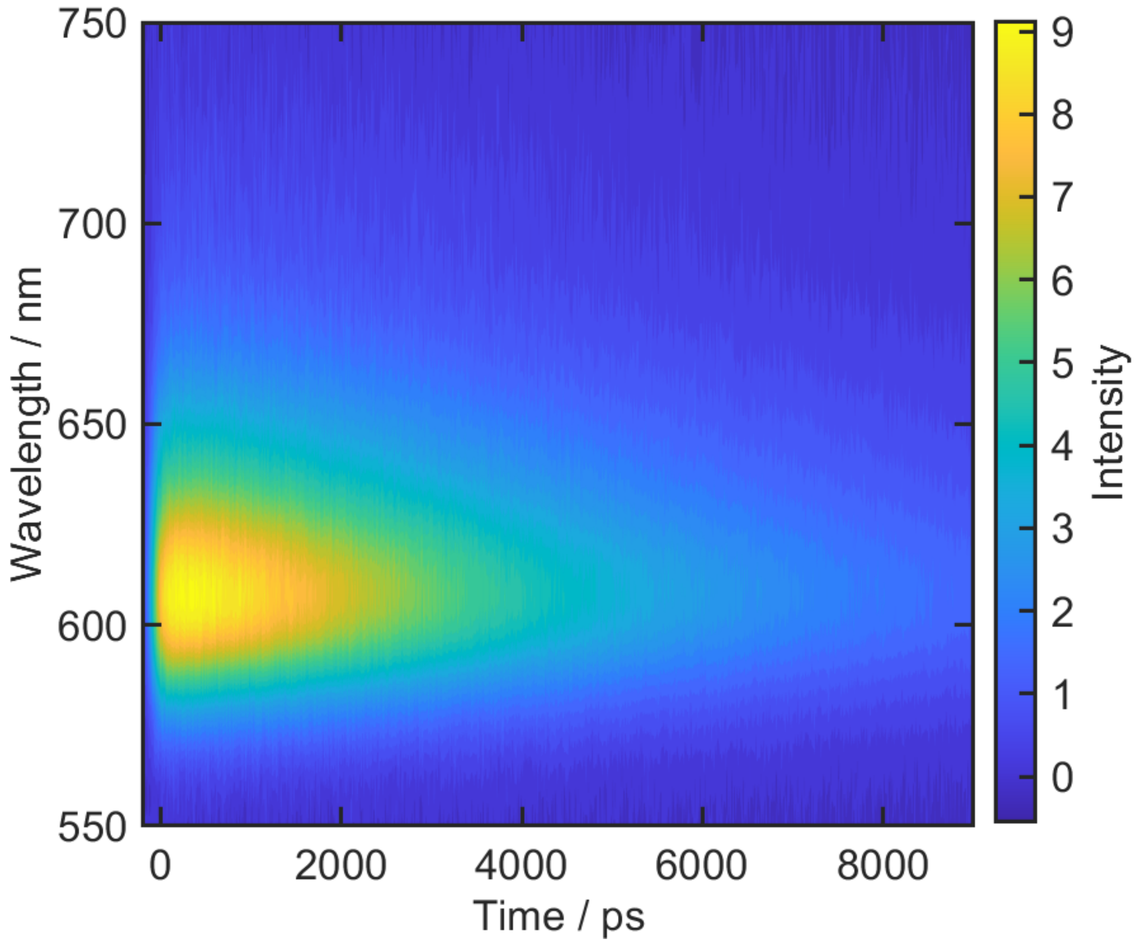

Figure S20. WR-TCSPC contour map of Nile Red in dichloromethane using 545 nm photoexcitation.

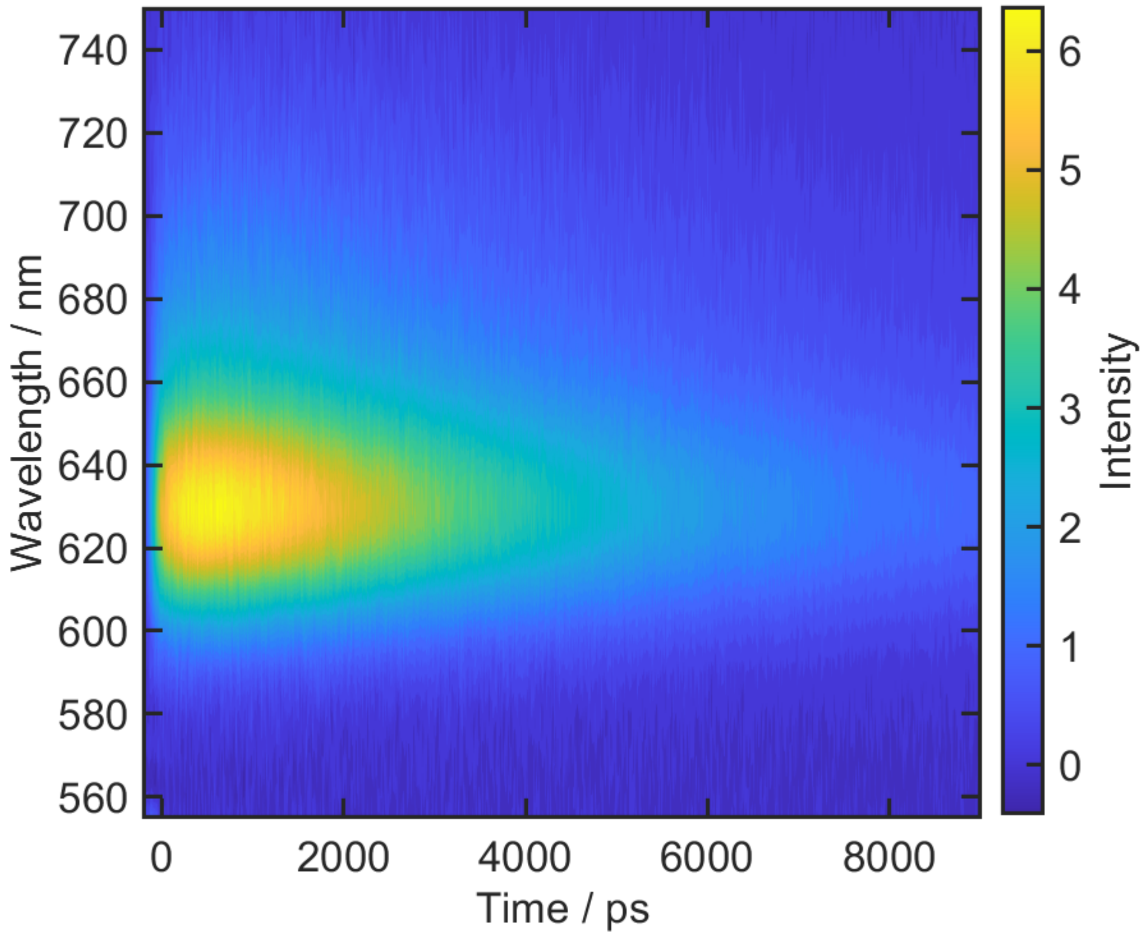

Figure S21. WR-TCSPC contour map of Nile Red in dimethyl sulphoxide using 550 nm photoexcitation.

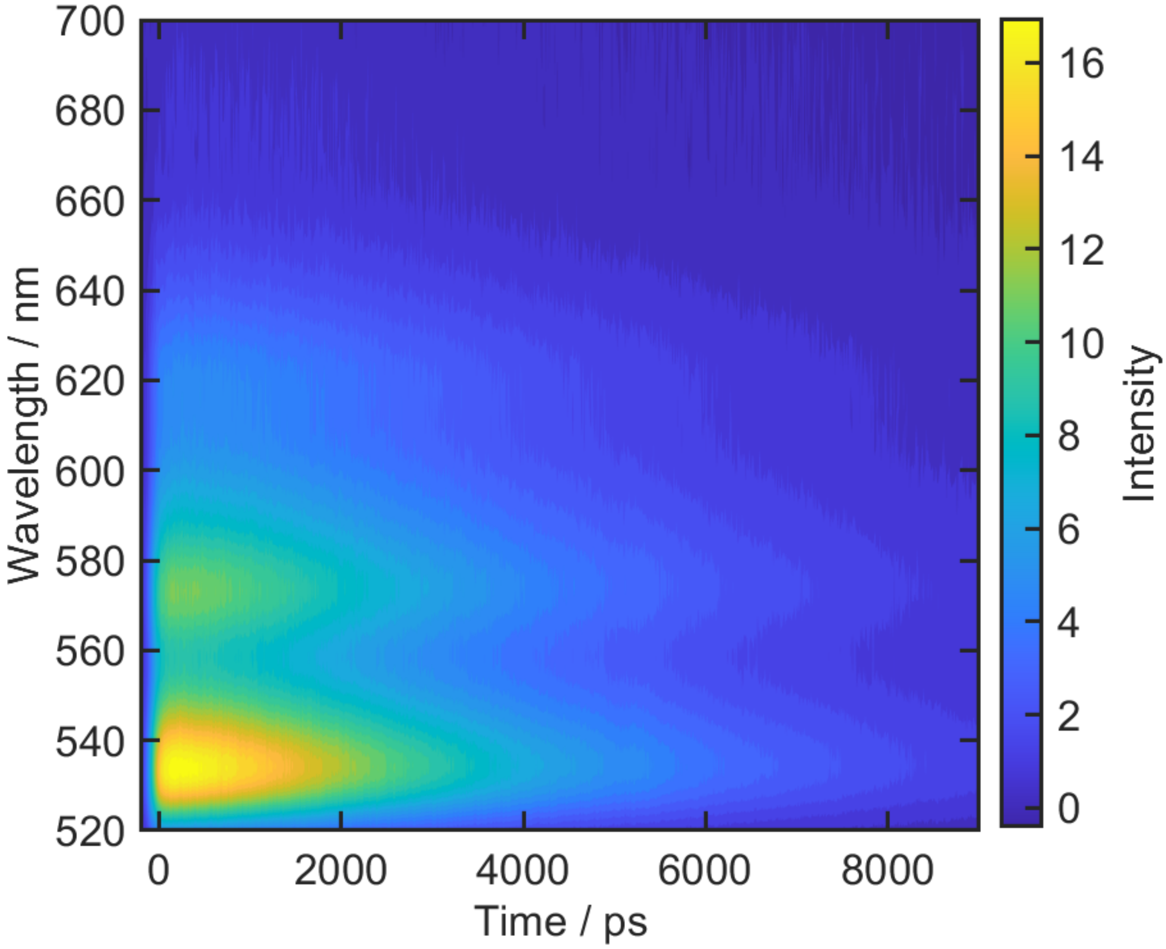

Figure S22. WR-TCSPC contour map of Nile Red in squalane using 420 nm photoexcitation.

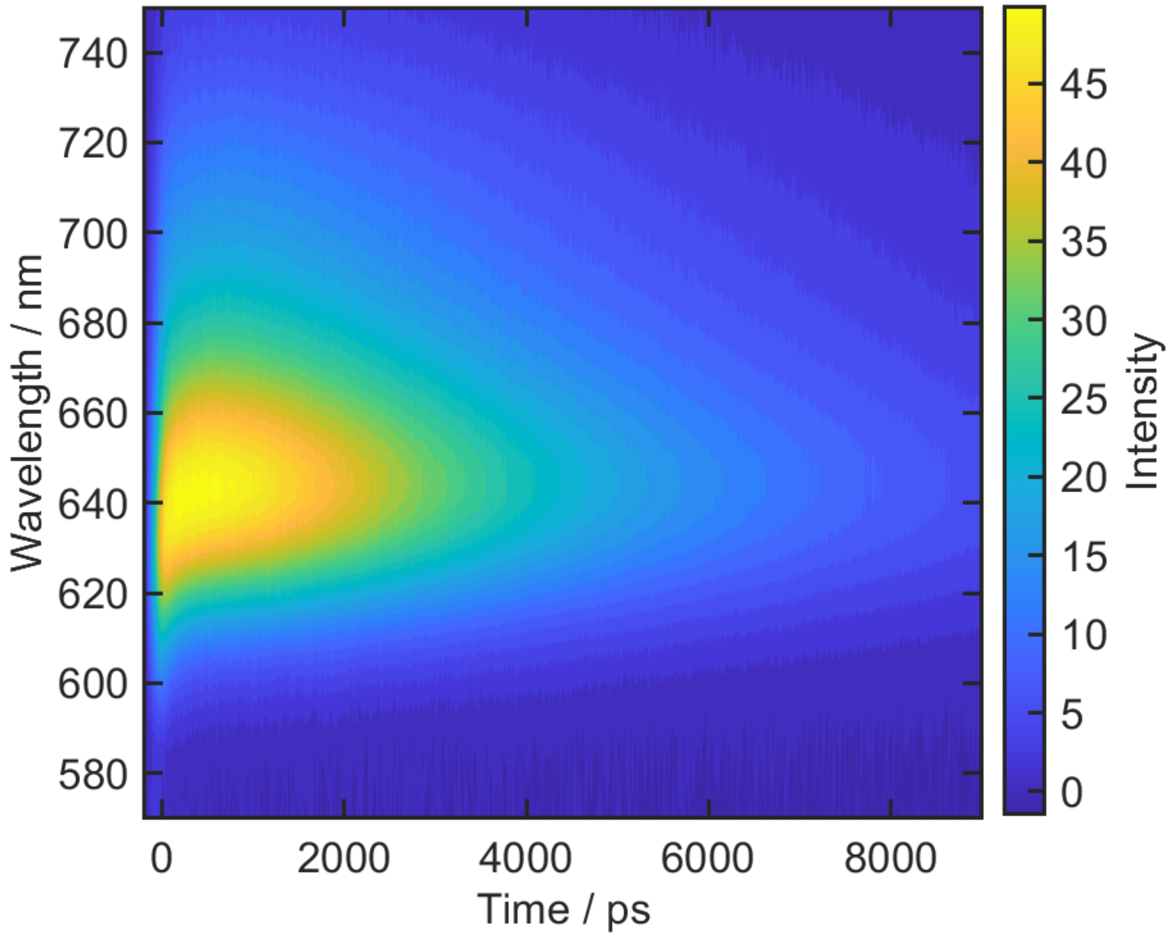

Figure S23. WR-TCSPC of Nile Red in benzyl alcohol using 570 nm photoexcitation.

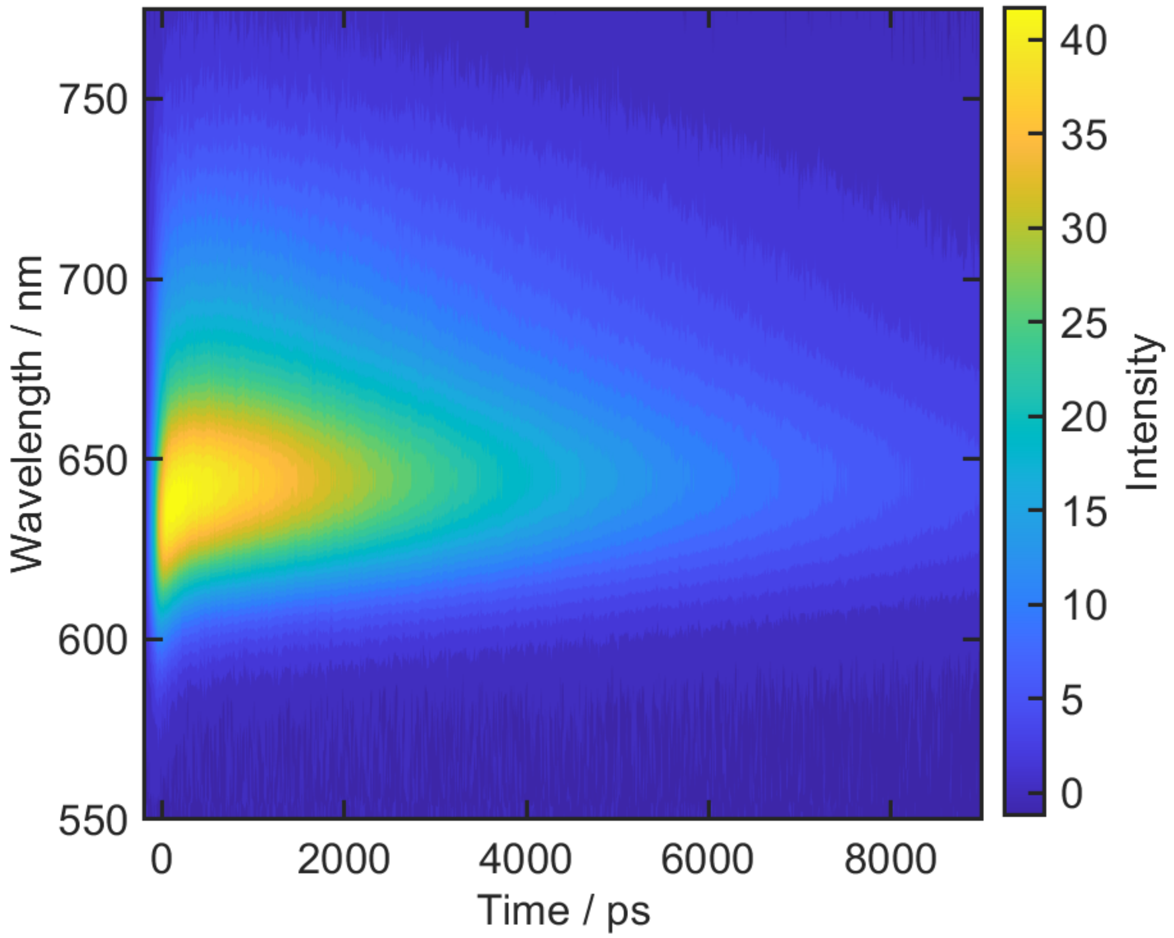

Figure S24. WR-TCSPC contour map of Nile Red in diethylene glycol using 560 nm photoexcitation.

7. Transient Absorption Spectroscopy Data

7.1 Dichloromethane

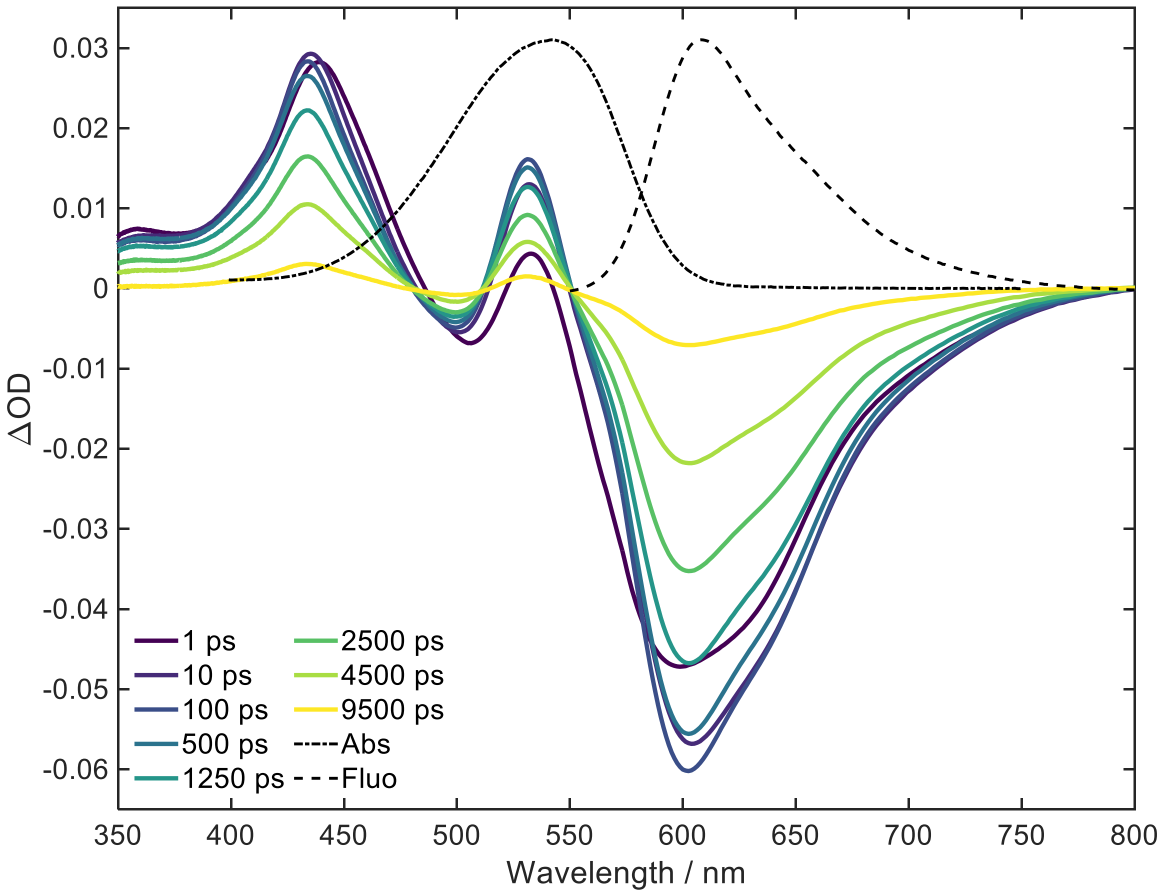

Figure S25. Transient Absorption of Nile Red in DCM excited with a pump wavelength of 545 nm.

7.2 Methanol

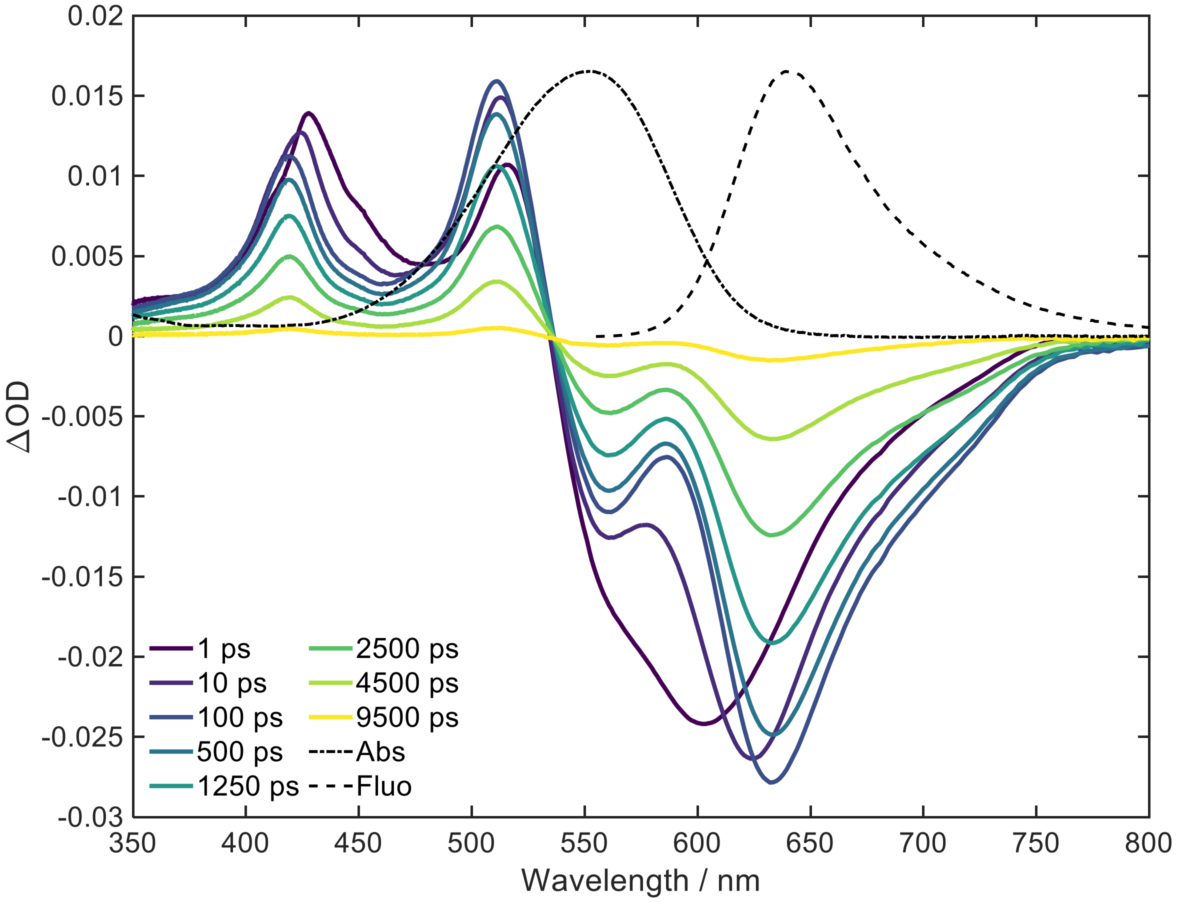

Figure S26. Transient Absorption of Nile Red in methanol excited with a pump wavelength of 553 nm.

7.3 Dimethyl Sulphoxide

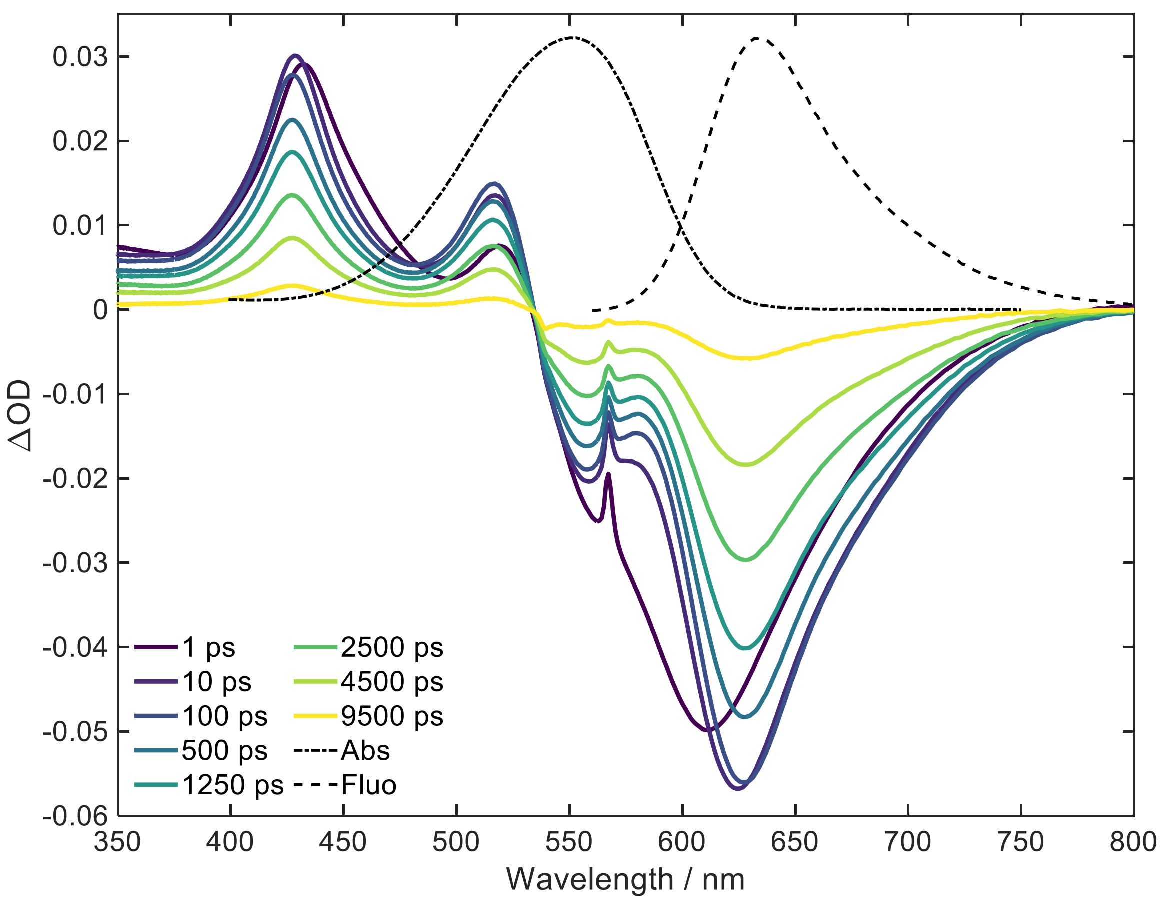

Figure S27. Transient Absorption of Nile Red in DMSO excited with a pump wavelength of 553 nm. At 553 nm there is a small amount of pump scatter that contaminates the TA signal.

7.4 Toluene

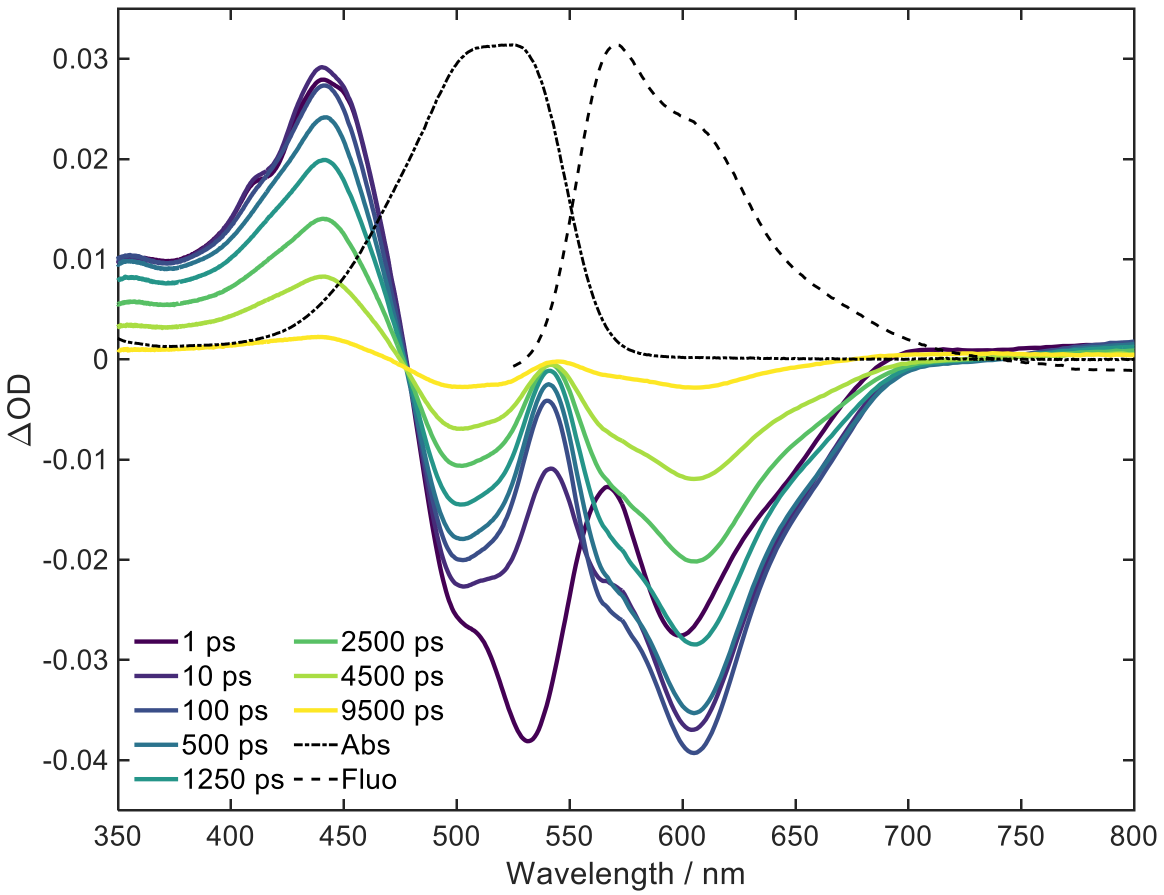

Figure S28. Transient Absorption of Nile Red in toluene excited with a pump wavelength of 530 nm.

8. Time-Resolved Infrared Spectroscopy Data

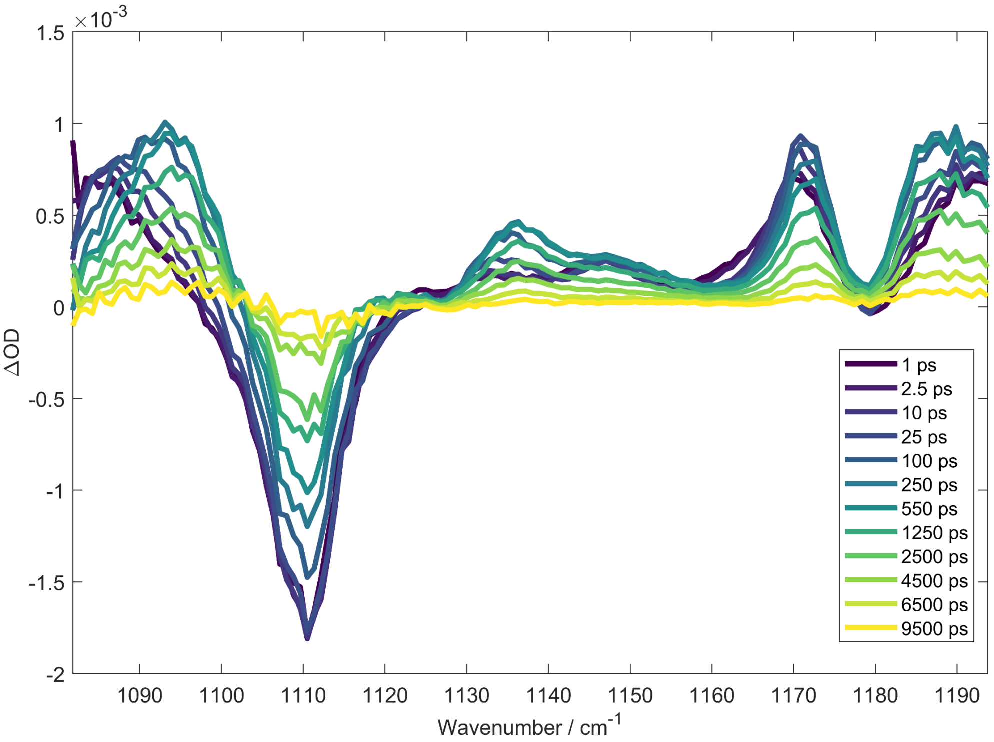

Figure S29. TRIR of Nile Red in 1-pentanol after 545 nm photoexcitation.

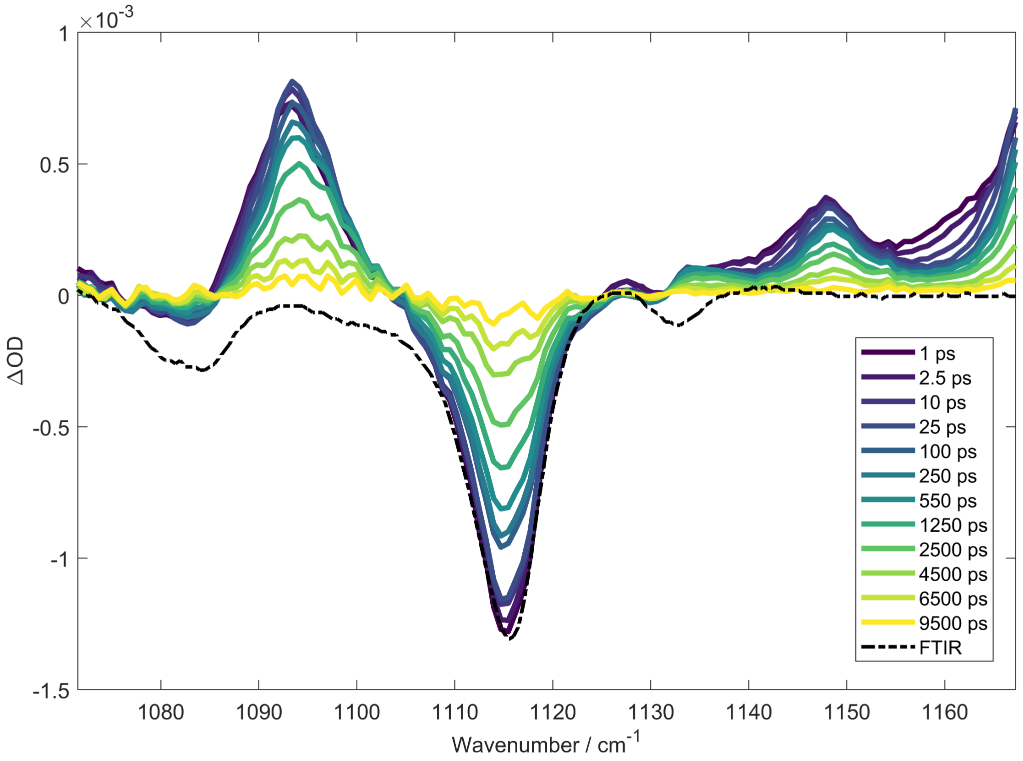

Figure S30. TRIR of Nile Red in DCM-d_2. FTIR overlaid (black solid line), inverted, and scaled to match intensity of the main bleach feature at 1115 cm^{-1}. Sample excited at a pump wavelength of 545 nm.

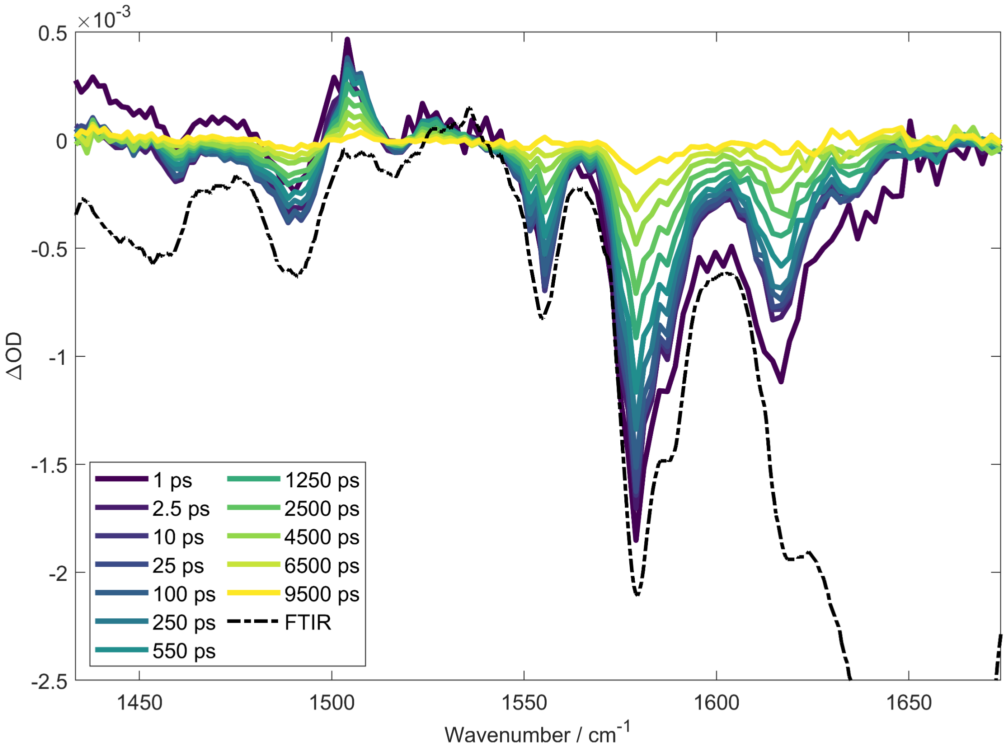

Figure S31. TRIR of Nile Red in DMSO-*d*$_6$. FTIR overlaid (black solid line), inverted, and scaled to match intensity of the main bleach feature at 1580 cm^{-1}. Sample excited at a pump wavelength of 553 nm.

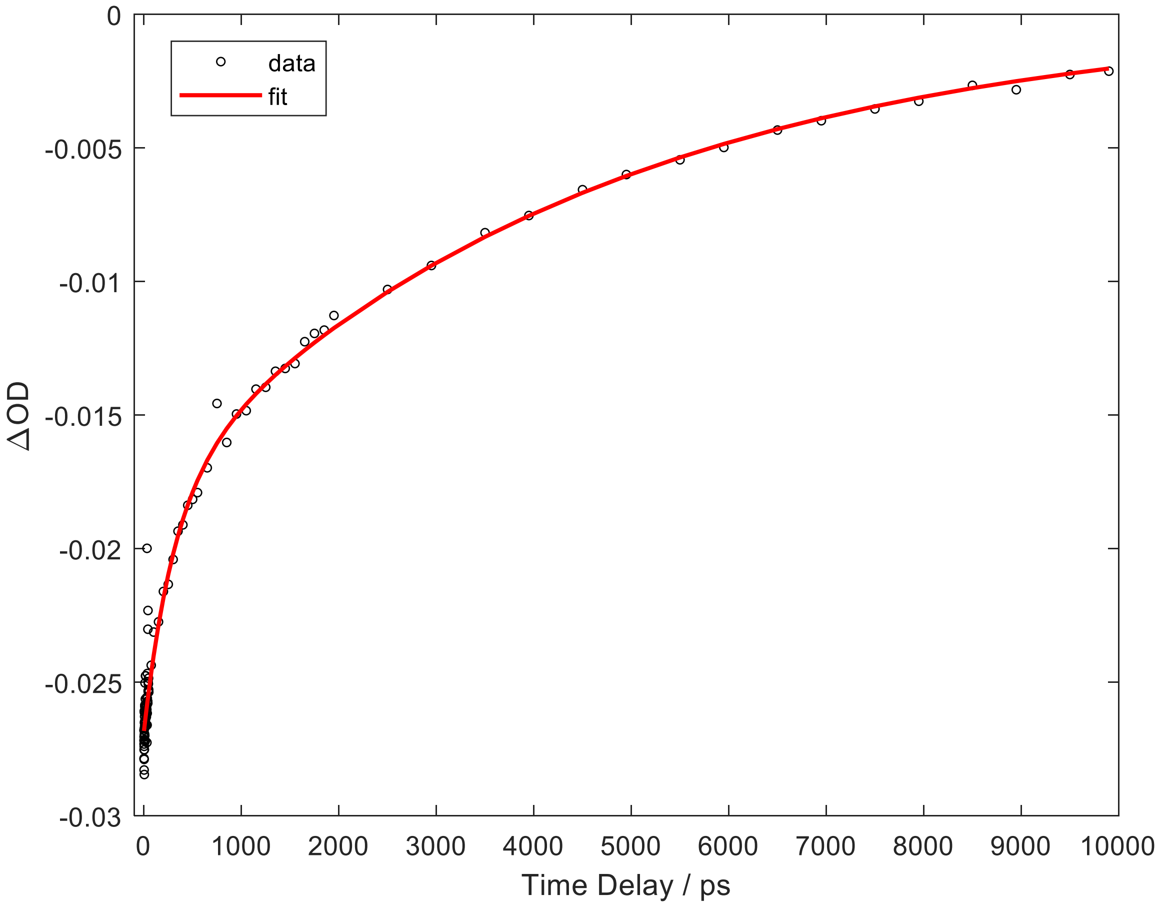

Figure S32. Kinetics GSB centred at ~1115 cm^{-1} from the TRIR of Nile Red in 1-pentanol.

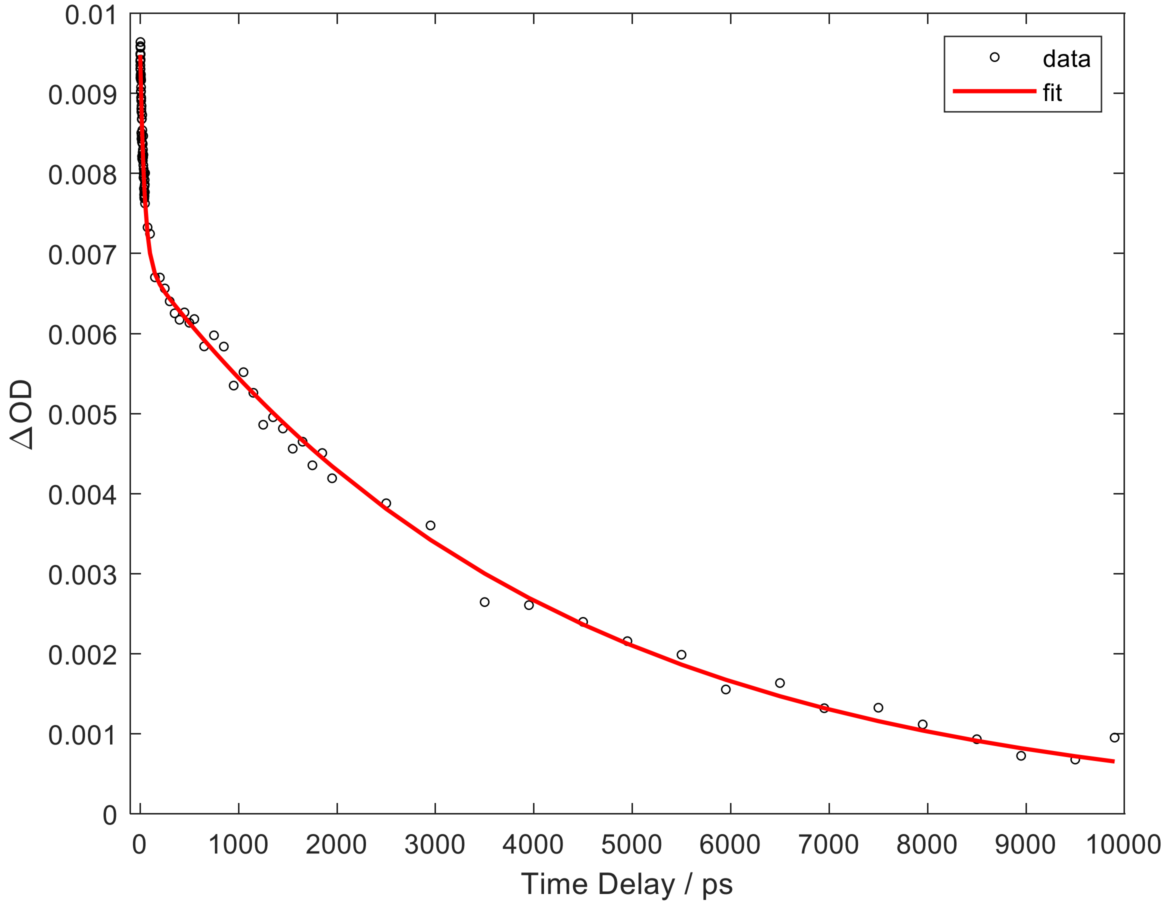

Figure S33. Kinetics of positive feature centred at ~1148 cm^{-1} from the TRIR of Nile Red in DCM-d_2.

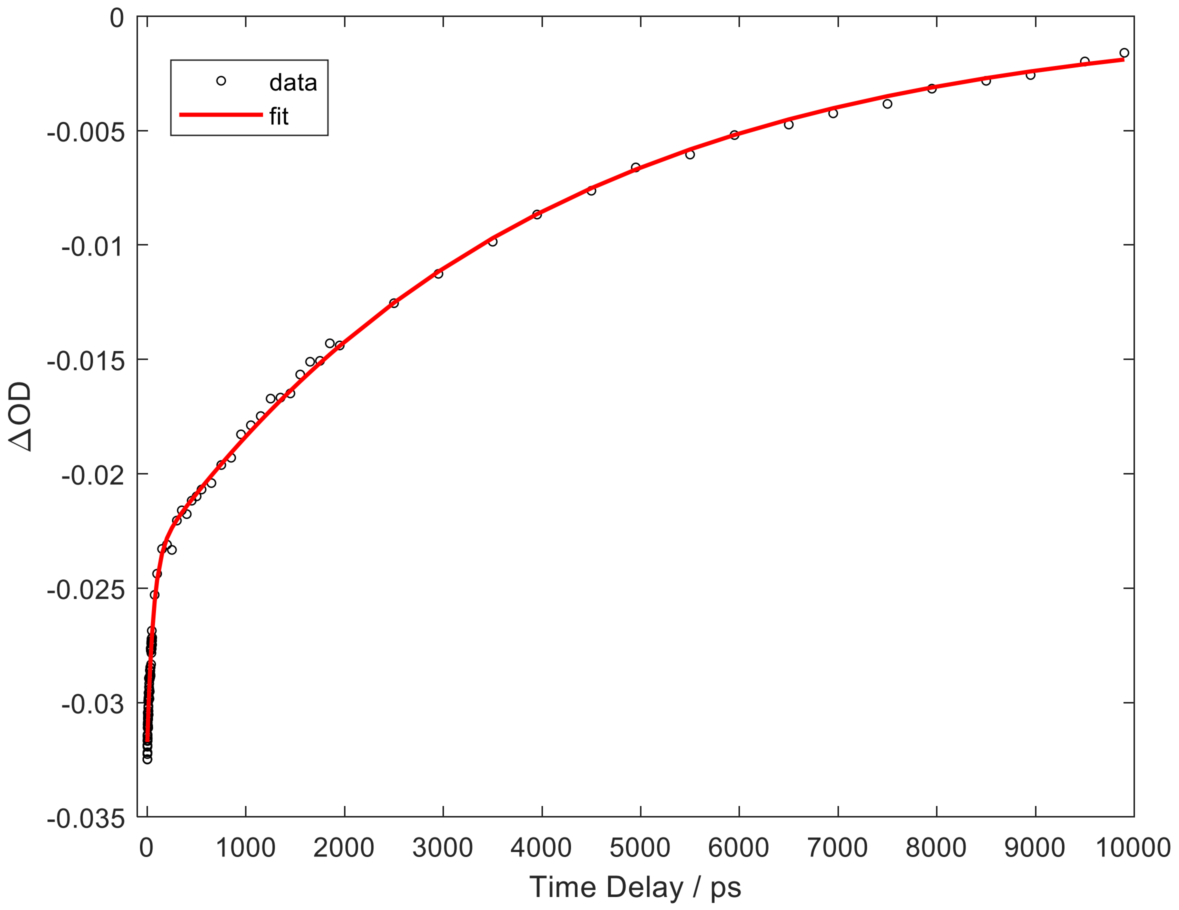

Figure S34. Kinetics GSB centred at ~1115 cm^{-1} from the TRIR of Nile Red in DCM-d_2.

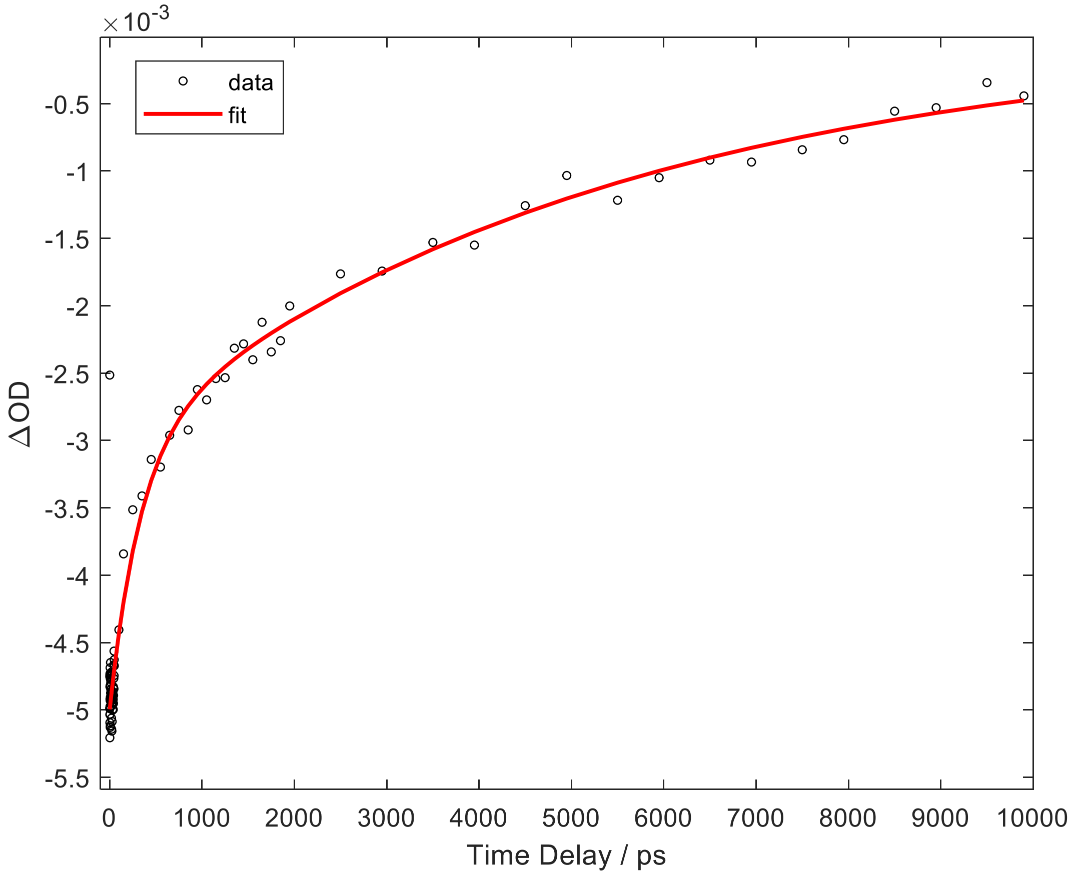

Figure S35. Kinetics GSB centred at ~1580 cm^{-1} from the TRIR of Nile Red in DMSO-d_6.

9. Summary of Kinetics extracted from TA and TRIR measurements

Table S3. Summary of the lifetimes extracted from TA and TRIR data for ground state bleach (GSB), excited state absorption (ESA) and stimulated emission (SE) features. Note that * denotes an exponential rise component.

Solvent	Feature	A_1 / %	A_2 / %	A_3/ %	τ_1 / ps	τ_2 / ps	τ_3 / ps
1-Pentanol	TRIR GSB	—	45	55	—	192 ± 27	3580 ± 344
	TA ESA2	*6	11	83	10 ± 3	185 ± 48	3867 ± 92
	TA SE	*11	12	77	11 ± 1	120 ± 19	3956 ± 45
DMSO	TRIR GSB	—	30	70	—	273 ± 38	4444 ± 207
	TA ESA2	*3	17	80	6 ± 3	236 ± 42	4194 ± 117
	TA SE	*15	8	77	5 ± 4	292 ± 63	4276 ± 75
DCM	TRIR GSB	—	26	74	—	60 ± 4	3917 ± 104
	TA ESA2	22	—	70	—	10 ± 4	4381 ± 87
	TA SE	*17	—	83	—	10 ± 1	3859 ± 111
Methanol	TA GSB	20	14	66	1.3 ± 0.2	16 ± 2	2920 ± 53
	TA ESA2	18	—	82	—	13 ± 1	2971 ± 55
	TA SE	13	—	87	—	16 ± 2	3048 ± 46
Toluene	TA GSB	18	12	70	2.0 ± 0.3	70 ± 14	3663 ± 195
	TA ESA2	*13	13	74	14 ± 4	85 ± 28	3762 ± 88
	TA SE	*22	1	77	8 ± 1	85 ± 28	3634 ± 66

10. Effect of Solvent Hydrogen–Bonding on Internal Conversion Pathway

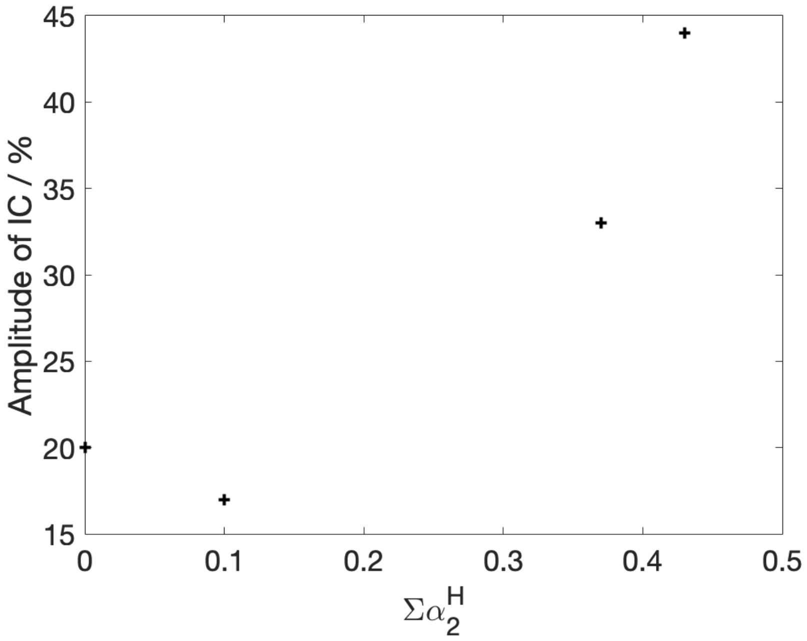

Figure S36. Correlation between solvent hydrogen-bonding (as quantified by Abraham's hydrogen-bonding acidity parameter $\Sigma\alpha_2^H$ [13]) and the relative amplitude of the internal conversion pathway determined by transient spectroscopies.

References

(1) Polak, D. W.; Hannon, A. D. P.; Marczak Giorio, G. A.; Hawkins, O. A.; Oliver, T. A. A. The Solvent-Dependent Photophysics of Diphenyloctatetraene. *The Journal of Physical Chemistry B* **2023**, *127* (38), 8199–8207. https://doi.org/10.1021/acs.jpcb.3c03737.

(2) Perri, A.; Gaida, J. H.; Farina, A.; Preda, F.; Viola, D.; Ballottari, M.; Hauer, J.; De Silvestri, S.; D'Andrea, C.; Cerullo, G.; Polli, D. Time- and Frequency-Resolved Fluorescence with a Single TCSPC Detector via a Fourier-Transform Approach. *Optics Express* **2018**, *26* (3), 2270. https://doi.org/10.1364/oe.26.002270.

(3) Greetham, G. M.; Sole, D.; Clark, I. P.; Parker, A. W.; Pollard, M. R.; Towrie, M. Time-Resolved Multiple Probe Spectroscopy. *Review of Scientific Instruments* **2012**, *83* (10), 103107. https://doi.org/10.1063/1.4758999.

(4) Greetham, G. M.; Donaldson, P. M.; Nation, C.; Sazanovich, I. V.; Clark, I. P.; Shaw, D. J.; Parker, A. W.; Towrie, M. A 100 kHz Time-Resolved Multiple-Probe Femtosecond to Second Infrared Absorption Spectrometer. *Applied Spectroscopy* **2016**, *70* (4), 645–653. https://doi.org/10.1177/0003702816631302.

(5) Casida, M. E. Time-Dependent Density Functional Response Theory for Molecules. In *Recent Advances In Density Functional Methods: (Part I)*; World Scientific, 1995; pp 155–192. https://doi.org/10.1142/9789812830586_0005.

(6) Chai, J.-D.; Head-Gordon, M. Long-Range Corrected Hybrid Density Functionals with Damped Atom–Atom Dispersion Corrections. *Physical Chemistry Chemical Physics* **2008**, *10* (44), 6615–6620. https://doi.org/10.1039/B810189B.

(7) Weigend, F.; Ahlrichs, R. Balanced Basis Sets of Split Valence, Triple Zeta Valence and Quadruple Zeta Valence Quality for H to Rn: Design and Assessment of Accuracy. *Physical Chemistry Chemical Physics* **2005**, *7* (18), 3297–3305. https://doi.org/10.1039/B508541A.

(8) Humphrey, W.; Dalke, A.; Schulten, K. VMD: Visual Molecular Dynamics. *Journal of Molecular Graphics* **1996**, *14* (1), 33–38. https://doi.org/10.1016/0263-7855(96)00018-5.

(9) Hättig, C.; Weigend, F. CC2 Excitation Energy Calculations on Large Molecules Using the Resolution of the Identity Approximation. *The Journal of Chemical Physics* **2000**, *113* (13), 5154–5161. https://doi.org/10.1063/1.1290013.

(10) TURBOMOLE V7.4.1 2019, a Development of University of Karlsruhe and Forschungszentrum Karlsruhe GmbH, 1989-2007, TURBOMOLE GmbH, since 2007; Available from http://www.turbomole.com.

(11) Guido, C. A.; Mennucci, B.; Jacquemin, D.; Adamo, C. Planar vs. Twisted Intramolecular Charge Transfer Mechanism in Nile Red: New Hints from Theory. *Physical Chemistry Chemical Physics* **2010**, *12* (28), 8016–8023. https://doi.org/10.1039/b927489h.

(12) Murov, S. *Properties of Solvents Used in Organic Chemistry*. Properties of Solvents Used in Organic Chemistry. http://murov.info/orgsolvents.htm#TABLE%202 (accessed 2024-05-22).

(13) Abraham, M. H. Scales of Solute Hydrogen-Bonding: Their Construction and Application to Physicochemical and Biochemical Processes. *Chemical Society Reviews* **1993**, *22* (2), 73–83. https://doi.org/10.1039/CS9932200073.
